# Tumor Biomarkers in Head and Neck Squamous Cell Carcinoma: From Etiology and Pathogenesis to Treatment Response—A Scoping Review

**DOI:** 10.3390/biomedicines14081842

**Published:** 2026-08-16

**Authors:** Cosmina-Diana Drăgan, Alexandra-Maria Marin, Oana Cezara Frasina-Vlad, Luminița Măruțescu, Petronela Ancuța, Anca Ionela Cîrstea, Mihai Dumitru Tudosie, Alexandru Nicolaescu, Catrinel Beatrice Simion-Antonie, Simona Andreea Rujan, Bianca Petra Taher, Daniel Voiculescu, Şerban Vifor Gabriel Berteşteanu, Raluca Grigore

**Affiliations:** 1 Faculty of Medicine, Carol Davila University of Medicine and Pharmacy, 050474 Bucharest, Romania; alexandra-maria.marin@drd.umfcd.ro (A.-M.M.); anca-ionela.cirstea@drd.umfcd.ro (A.I.C.); mihai-dumitru.tudosie@drd.umfcd.ro (M.D.T.); alexandru.nicolaescu@umfcd.ro (A.N.); catrinel.simion-antonie@drd.umfcd.ro (C.B.S.-A.); simona-andreea.rujan@drd.umfcd.ro (S.A.R.); bianca-petra.taher@drd.umfcd.ro (B.P.T.); daniel.voiculescu@umfcd.ro (D.V.); serban.bertesteanu@umfcd.ro (Ş.V.G.B.); 2 Department of ENT, Head and Neck Surgery, Colţea Clinical Hospital, 030171 Bucharest, Romania; 3 Faculty of Biology, University of Bucharest, 050663 Bucharest, Romania; vlad.oana18@gmail.com (O.C.F.-V.); luminita.marutescu@bio.unibuc.ro (L.M.);; 4 Department of Microbiology, Infectious Diseases and Immunology, Faculty of Medicine, Université de Montréal, Montréal, QC H3C 3J7, Canada; 5 Centre de Recherche du Centre Hospitalier de l’Université de Montréal (CRCHUM), Montréal, QC H2X 0A9, Canada; 6 Department of ENT, Head and Neck Surgery, Emergency University Hospital, 021373 Bucharest, Romania; 7 Department of ENT, Prof. Dr. Dimitrie Gerota Emergency Hospital, 021373 Bucharest, Romania; 8 Department of Surgery I, Emergency University Hospital, 021373 Bucharest, Romania

**Keywords:** biomarker, cancer, prognostic, diagnostic, predictive, tumor, treatment, carcinoma

## Abstract

Currently, the global incidence of head and neck squamous cell carcinoma (HNSCC) continues to increase, with a large proportion of cases being diagnosed at advanced stages. Conventional oncological treatments are often associated with poor quality of life, while the aggressive biological behavior of these tumors results in recurrence in approximately half of treated patients according to current evidence. The need for rapid, targeted diagnosis and personalized treatment has driven research toward the identification of tumor biomarkers with diagnostic, prognostic, and predictive value for treatment response. In this scoping review, we performed a comprehensive literature search to map the available evidence on tumor biomarkers in HNSCC. We identified a wide range of biomarkers, several of which exhibit overlapping roles, and classified them according to their genomic, proteomic, cytokine-related, tumor microenvironment, metabolic, and microbiota-associated characteristics. This scoping review also summarizes the current evidence regarding biomarkers involved in carcinogenesis, treatment response and therapy resistance, while highlighting their current level of clinical applicability. Although only a limited number of biomarkers have currently been implemented in routine clinical practice in head and neck cancers, the studies included in this review identified several promising candidates with the potential to be incorporated into clinical practice following further prospective validation and methodological standardization. Many additional biomarkers are still under investigation, opening new perspectives for future diagnostic and therapeutic targets and supporting the development of personalized multimodal treatment strategies for patients with HNSCC.

## 1. Introduction

Head and neck cancer constitutes the seventh most prevalent malignancy worldwide, encompassing a broad spectrum of neoplasms arising within the upper aerodigestive tract, salivary glands, skin and temporal bone [1]. Across many regions worldwide, the burden of HNSCC is showing an upward trajectory, with a disproportionate increase observed in younger cohorts. Current epidemiological models anticipate that overall incidence could rise by approximately 30% by the year 2030 [2]. As a histopathological subtype, squamous cell carcinomas of the head and neck originating from the mucosal lining epithelium are encountered with the highest frequency, accounting for approximately 90% of cases [3]. This type of cancer develops predominantly in elderly smokers and chronic alcohol consumers and is epidemiologically prevalent in regions associated with betel nut use. Tobacco use represents the principal etiological risk factor implicated in the development of head and neck cancers, while its concomitant association with alcohol consumption exerts a synergistic effect, mediated by ethanol-induced enhancement of mucosal permeability to carcinogenic agents [3]. Viral infections with human papillomavirus and Epstein–Barr virus are recognised as carcinogenic factors for developing HNC, are more frequently encountered in younger patients without significant exposure to the previously mentioned risk factors, and are associated with long-term prognosis [3,4]. Other risk factors described in the literature are genetic predisposition, hereditary cancer syndromes, immunodeficiency, chronic inflammation of the oral cavity, a history of radiotherapy or exposure to high doses of ionizing radiation, and exposure to occupational carcinogens [5]. Management of HNSCC relies on a multimodal therapeutic strategy that may include surgery, radiotherapy, chemotherapy, targeted agents, and immune checkpoint inhibition, often used in combination, reflecting the fact that around 60% of patients are diagnosed at an advanced stage or with metastatic disease [6]. Even with regular follow-up and individualized treatment, advanced-stage disease remains associated with significant mortality, with 5-year relative survival rates of approximately 60% and disease recurrence, as well as the development of metastases, occurring in up to 50% of patients within three years [2].

Long-term survivors often experience persistent treatment-related morbidity, which can adversely impact functional capacity, health-related quality of life, and overall mortality unrelated to cancer. Consequently, the identification of biological markers that reflect tumor behavior and predict therapeutic sensitivity has become increasingly important, as such tools could guide individualized treatment planning. This has driven substantial research efforts aimed at discovering reliable diagnostic, predictive and prognostic biomarkers in HNSCC [7].

Tumor biomarkers are described by the U.S. National Cancer Institute as proteins that are produced in high amounts by cancerous cells or other cells of the body in response to neoplasia. This includes studying biological specimens such as serum, plasma, saliva, tumor tissue, or other bodily fluids that can provide information regarding the etiology, pathogenesis, and prognosis of the diagnosed malignancy [8]. Tumor tissue biopsy remains the gold standard for the diagnosis of HNSCC; however, in order to guide oncological treatment selection, testing of specific biomarkers may be performed, particularly in cases where the tumor is surgically unresectable or considered inoperable due to patient comorbidities.

At present, only a limited number of diagnostic biomarkers have been validated for use in clinical practice, with some exhibiting specificity for particular neoplastic tissues. In HNSCC, biomarkers are commonly classified into diagnostic, prognostic, and predictive categories based on their clinical utility. Diagnostic biomarkers are used to detect and confirm the presence of disease and may also assist in tumor characterization. Prognostic biomarkers provide information regarding the likely natural course of the disease, including the risk of recurrence and overall survival, independent of therapeutic intervention. In contrast, predictive biomarkers are associated with the likelihood of response to a specific treatment modality and are therefore essential for guiding therapeutic decision-making. Certain biomarkers in HNSCC may demonstrate overlapping roles, exhibiting diagnostic, prognostic and predictive significance depending on the clinical context. Nevertheless, the field of tumor biomarkers still requires further research, as the interaction between the host, the tumor microenvironment, and the tumor itself remains insufficiently investigated. The available literature encompasses a wide range of biomarkers with different biological characteristics and potential clinical applications across various anatomical subsites of HNSCC. This heterogeneity highlights the need to comprehensively map the available evidence, identify promising biomarkers, and highlight current research gaps. Therefore, a scoping review approach was considered appropriate to summarize the available evidence and characterize the potential clinical applications of tumor biomarkers in HNSCC. Therefore, the routine implementation of biomarkers in oncology could play a key role in refining therapeutic decision-making, allowing clinicians to personalize treatments and improve treatment effectiveness and overall standards of care, thereby contributing to the development of precision medicine [2].

The aim of this scoping review was to map and summarize the current evidence regarding tumor biomarkers in HNSCC, identify the biomarker categories investigated, describe their potential clinical applications, and highlight current research gaps. The review also characterized the current level of clinical applicability of the identified biomarkers, distinguishing biomarkers currently used in clinical practice from those that remain investigational.

## 2. Materials and Methods

### 2.1. Registration of Protocols and Study Design

The research question was formulated in accordance with the PICo framework for qualitative research [9]. The Population (P) comprised patients diagnosed with head and neck malignancies, with particular emphasis on HNSCC. The Interest (I) encompassed tumor biomarkers, including diagnostic, prognostic, and predictive biomarkers, with relevance to disease detection, biological behavior, and treatment response. The Context (Co) included the investigation of these biomarkers in relation to carcinogenesis, etiological factors, the molecular and cellular mechanisms of pathogenesis, and their role in therapeutic stratification and clinical outcomes.

### 2.2. Eligibility Criteria

Inclusion criteria were established at the beginning of the review process and included recently published articles (within the past five years) to ensure up-to-date evidence, being written in English, available in full-text format, published in peer-reviewed journals, conducted on human subjects, and focused on the investigation of validated tumor biomarkers for diagnosis, prognosis and treatment of HNSCC, as well as emerging biomarkers. In the selected studies, the role of tumor biomarkers was assessed in at least one of the following domains: etiology, pathogenesis, diagnosis, treatment guidance and selection of optimal therapeutic strategies, as well as in the prediction of long-term treatment response in head and neck cancers.

The following exclusion criteria were applied: articles older than five years (in order to ensure that the review reflects the most recent evidence), publications written in languages other than English, studies with restricted access, research conducted on animal models or in vitro systems, articles not addressing head and neck cancers, and studies focusing on histopathological subtypes other than squamous cell carcinoma. Conference abstracts, editorials, and letters to the editor were also excluded.

### 2.3. Search Strategy

This review was conducted in accordance with the PRISMA Extension for Scoping Reviews (PRISMA-ScR) [10]. Supplementary Table S1 contains the PRISMA checklist for the scoping review. The study was designed as a scoping review of the literature, aiming to synthesize current evidence on tumor biomarkers in HNSCC. Due to the heterogeneity of biomarkers, study designs, and reported outcomes, a narrative synthesis approach was adopted.

A comprehensive structured literature search was performed, and studies were selected based on predefined inclusion and exclusion criteria in the PubMed global database, including different types of reviews and articles. The following keywords were used: “head and neck cancer”, “biomarkers”, “diagnostic”, “prognostic”, and “predictive response”. The research protocol began on 1 May 2026.

The search was performed using a combination of Medical Subject Headings (MeSH) terms and terms related to head and neck neoplasms and tumor biomarkers. The search strategy incorporated terms addressing diagnostic, prognostic, and predictive biomarkers, as well as etiology, pathogenesis, and treatment response, which were combined using Boolean operators (AND, OR).

### 2.4. Data Collection

We used three different search syntaxes within the PubMed database in order to identify diagnostic, prognostic, and predictive biomarkers for treatment response, as follows: (“head and neck neoplasms”[MeSH Terms] OR “head and neck cancer” OR “HNSCC” OR “head and neck squamous cell carcinoma”) AND (“biomarkers”[MeSH Terms] OR “tumor markers” OR “molecular biomarkers”) AND (“diagnosis”[MeSH Terms] OR “diagnostic biomarkers” OR “early detection of cancer”[MeSH Terms]) for identifying studies about diagnostic biomarkers; (“head and neck neoplasms”[MeSH Terms] OR “head and neck cancer” OR “HNSCC” OR “head and neck squamous cell carcinoma”) AND (“biomarkers”[MeSH Terms] OR “tumor markers” OR “molecular biomarkers”) AND (“prognosis”[MeSH Terms] OR “prognostic biomarkers” OR “survival analysis”[MeSH Terms] OR “disease-free survival” OR “overall survival”) for identifying studies about prognostic biomarkers; (“head and neck neoplasms”[MeSH Terms] OR “head and neck cancer” OR “HNSCC” OR “head and neck squamous cell carcinoma”) AND (“biomarkers”[MeSH Terms] OR “tumor markers” OR “molecular biomarkers”) AND (“treatment outcome”[MeSH Terms] OR “treatment response” OR “predictive biomarkers” OR “therapy response” OR “drug response”) for identifying studies about biomarkers predictive of treatment response.

**Figure 1:**
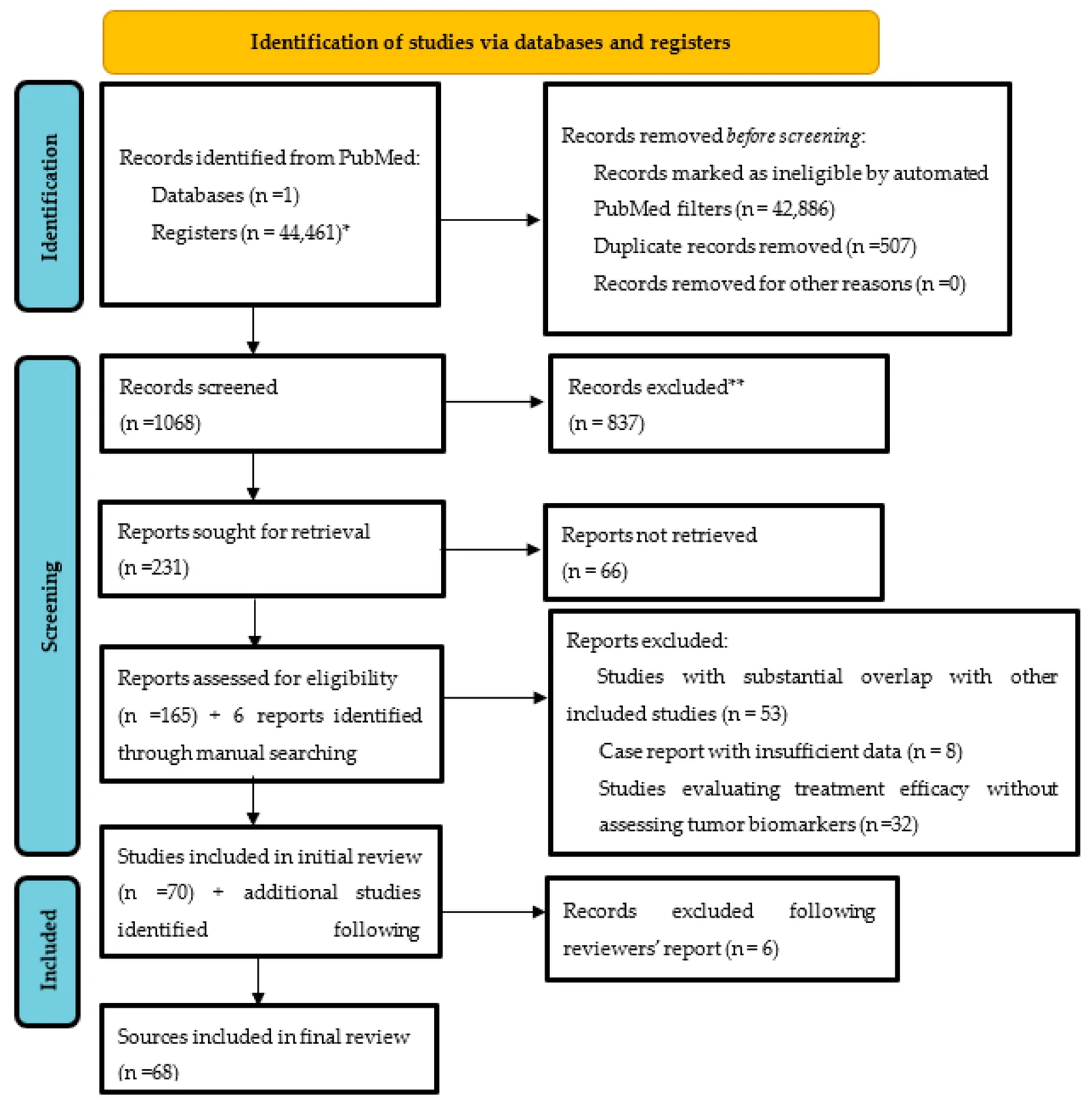
PRISMA-ScR flow diagram of the research. * Number of records identified from each register searched: 28,325 results for diagnostic biomarkers, 12,635 results for prognostic biomarkers and 3501 results for predictive biomarkers, respectively. ** Of the 837 records excluded during the screening stage, 771 were excluded based on the predefined automated filtering criteria, while 66 were excluded by the authors during title and abstract screening.

The selection methodology is presented in Figure 1, following the PRISMA-ScR flow diagram framework.

Our search identified 28,325 records using the diagnostic biomarker search strategy, 12,635 records using the prognostic biomarker search strategy and 3501 records using the predictive biomarker search strategy. In total, 44,461 records were retrieved across the three search strategies, covering the period from 1976 to 2026. No dedicated automation software was used for study screening. Automated filtering was performed using the built-in PubMed filters according to the predefined eligibility criteria, including publication date (last five years), English language, free full-text availability, human studies, and article type. Application of the predefined PubMed filters resulted in the exclusion of 42,886 records (35,269 published more than five years ago, 3199 records that were not free full text, 4348 records with publication types outside the scope of this review, 36 records that were not written in English and 34 records that were not conducted on human subjects). Following the application of the predefined PubMed filters, 507 duplicate records resulting from overlap between the three search strategies were identified and removed using Zotero (version 9.0.5). Zotero was used exclusively for reference management, duplicate detection, and organization of the retrieved records. The remaining records were then managed in Zotero, where the software’s search function was used to facilitate the identification of records containing predefined keywords relevant to the eligibility criteria. During the initial screening stage, 837 records were excluded based on the predefined eligibility criteria, including records focusing on non-head and neck cancers or on histopathological subtypes other than HNSCC. Two independent authors (C.-D.D. and A.-M.M.) screened the titles and abstracts of the remaining records for eligibility according to the predefined inclusion and exclusion criteria, excluding an additional 66 records. All exclusions were subsequently verified by both authors to minimize the risk of erroneous exclusions. Any disagreements were resolved through discussion and consensus. Following screening, 231 reports were sought for full-text assessment, of which 66 could not be retrieved. The remaining 165 reports were assessed for eligibility. Following the initial literature search, 6 additional relevant studies were identified through complementary manual searching and were assessed for eligibility. At the eligibility stage, 53 studies were excluded due to substantial overlap with other included studies, 8 case reports were excluded due to insufficient data, 8 articles were excluded because they were outside the scope of this review, and 32 studies were excluded because they evaluated treatment efficacy without assessing tumor biomarkers. Therefore, 70 sources were included following the initial selection process. Following the reviewers’ comments, 6 studies related to Epstein–Barr virus (EBV) were subsequently removed from the review, while 4 additional studies addressing oral microbiomics were incorporated. Thus, the final evidence base comprised 68 sources.

The characteristics of the included studies and reviews, including study design, population, tumor subsite, investigated biomarkers, and reported outcomes, are summarized in Supplementary Table S2.

We acknowledge that GenAI was used in this section of the manuscript to assist in formatting the search syntax for the predefined database search strategy, while all methodological decisions related to the search strategy were made exclusively by the authors.

### 2.5. Methodological Approach

Due to the methodological heterogeneity of the included literature, encompassing a wide range of tumor biomarkers, study designs and reported outcomes, a formal critical appraisal or risk of bias assessment using a single standardized tool was not performed, as this is not a mandatory component of scoping reviews and the primary objective was to map the available evidence rather than to evaluate the certainty of the evidence or estimate the effect of a specific intervention or diagnostic test. Accordingly, all eligible studies were included to ensure a comprehensive synthesis of the available evidence on tumor biomarkers in HNSCC. Disagreements were resolved by consensus.

## 3. Results

The aim of personalized medicine and the identification of reliable tumor biomarkers are to enable early detection of carcinogenesis risk and to classify tumors according to their molecular characteristics, thereby facilitating the development of diagnostic tests that are sensitive, specific, cost-effective, and reproducible [8].

The methods used for the detection of tumor biomarkers are diverse and include electron microscopy (for ultrastructural visualization of cellular components), immunohistochemistry (for the detection of antigens in paraffin-embedded tumor specimens), gene amplification using polymerase chain reaction, molecular hybridization (to detect complementary sequences in DNA–RNA hybrid molecules), DNA sequencing (to identify specific nucleotide sequences corresponding to tumor markers), fluorescent immunoassays (based on the detection of light emitted following the binding of labeled antibodies), CRISPR-based techniques for the rapid identification of specific nucleic acids (such as ctDNA, microRNA, and exosomes), and liquid biopsy approaches for the analysis of tumor biomarkers in biological fluid samples [8].

Scientific advances driven by the development of multiple detection methods have enabled a more rigorous classification of neoplasms, the implementation of targeted oncologic therapies, and the assessment of long-term prognosis. In this section, we provide a comprehensive stratification of tumor biomarkers identified in HNSCC within our analysis, according to their roles in diagnosis, prediction of treatment response, and prognosis.

### 3.1. Genomics

#### 3.1.1. Human Papillomavirus DNA Load and p16INK4a Suppressor Protein

The prevalence of HPV involvement in HNSCC is estimated to be around 33%, with rates reaching up to 80% in oropharyngeal cancers [10,11]. The 8th edition of the AJCC TNM classification introduced a major revision in the staging of OPSCC (oropharyngeal squamous cell carcinoma) by establishing separate staging systems for HPV-associated and HPV-independent tumors [12]. The National Comprehensive Cancer Network (NCCN) guidelines recommend HPV testing in the diagnosis of oropharyngeal cancers using analysis of E6/E7 oncogene microRNA by RT-PCR and ISH, in association with p16 IHC. HPV testing for other head and neck cancer subsites is not routinely recommended due to the relatively low contribution of the virus to carcinogenesis in these locations [13]. HPV types 6 and 11 are primarily responsible for the development of recurrent respiratory papillomatosis, whereas HPV16 is predominantly associated with a subset of HNSCC [14]. HPV attaches to the single-layered epithelium of the palatine tonsillar crypts, establishing infection through evasion of virus-specific T-cell responses, particularly those of cytotoxic T lymphocytes responsible for eliminating viral particles [15]. Oncogenesis occurs through persistent infection and integration of the HPV genome into the host cell genome, leading to overexpression of the viral oncogenes E6 and E7. E6 promotes degradation of the tumor suppressor protein p53 and inhibits apoptosis, while E7 inactivates the retinoblastoma protein (pRb), resulting in the release of E2F transcription factors and sustained uncontrolled cellular proliferation.

p16INK4a is a tumor suppressor protein encoded by the CDKN2A gene that slows down cell division, acting as a barrier to carcinogenesis by inhibiting cycline kinase 4. Overexpression of p16INK4a serves as a surrogate biomarker of HPV-associated disease, resulting from a feedback mechanism driven by altered transcriptional activity of the cell cycle [16]. Although p16INK4a IHC demonstrates high sensitivity for detecting HPV-associated OPSCC, its limited specificity may lead to an increased rate of false-positive results, particularly in populations where the prevalence of HPV-positive tumors is low [16]. This may negatively impact patient stratification and eligibility and could compromise the accuracy of HPV detection in non-oropharyngeal subsites, where the reported prevalence of HPV-associated disease is generally low [16].

As previously mentioned, the majority of HPV-associated HNSCCs are OPSCC. Patients with HPV-related oropharyngeal squamous cell carcinoma are generally diagnosed at a younger age and often lack significant exposure to traditional risk factors such as tobacco use. They typically present with fewer comorbidities and show improved treatment responses and survival outcomes [17]. According to the AJCC 8th edition TNM staging system for OPSCC in HPV-associated tumors, the presence of cervical lymph node metastases does not confer the same adverse prognostic significance observed in HPV-negative disease; therefore, the nodal classification was simplified and the overall stage grouping was downstaged in many cases. Consequently, patients previously classified as stage IV under earlier editions may now be categorized as stage I or II if their tumors are p16-positive. In contrast, HPV-negative OPSCC continues to follow the traditional TNM stratification, including extranodal extension as an important adverse prognostic factor [12]. Currently, despite differences in tumor staging based on HPV-driven stratification (and the associated more favorable prognosis), standard oncologic treatment does not differ between the two entities. Consequently, younger patients remain exposed to the risk of developing secondary malignancies induced by radiotherapy. However, several clinical trials investigating treatment de-escalation strategies are currently ongoing [18].

Patients who exhibit p16INK4a-positive tumors in the absence of detectable HPV infection have significantly worse clinical outcomes compared to patients with concurrent p16 and HPV positivity. This finding raises important concerns regarding the use of p16 as an independent marker for treatment de-escalation, as p16-positive/HPV-negative patients, who may have an increased risk of recurrence, could be inappropriately considered for less intensive therapeutic approaches [19]. Pouso et al. conducted a meta-analysis demonstrating that p16INK4a is associated with a negative prognostic impact on the progression of oral epithelial dysplasia to oral cavity squamous cell carcinoma [20].

HPV status in HNSCC has been investigated as a potential predictive biomarker for response to immune checkpoint inhibitors (ICIs), with HPV-positive tumors generally showing increased immunogenicity and higher PD-L1 expression. Clinical studies have reported higher response rates to PD-1/PD-L1 blockade in HPV-positive patients, including early evidence from KEYNOTE-012, although these findings were not consistently confirmed in later trials such as KEYNOTE-040 and other studies with different ICIs. The variability in outcomes suggests that treatment response is influenced by additional factors, including smoking status and tumor mutational burden. Therefore, HPV positivity alone is not a reliable predictor of immunotherapy response and should be interpreted alongside other molecular and clinical biomarkers [2].

HPV-positive tumors, unlike HPV-negative head and neck malignancies, predominantly preserve wild-type TP53 status and demonstrate increased sensitivity to radiotherapy. Impairment of p53 signaling has generally been associated with the development of radioresistance, whereas radiosensitive tumor cells tend to exhibit higher baseline expression levels of p53. Nevertheless, despite the HPV-mediated functional degradation of p53 through viral oncoproteins, HPV-positive tumors paradoxically retain a more favorable radiosensitive phenotype compared with their HPV-negative counterparts [21].

Additionally, circulating tumor HPV DNA (ctHPV DNA) is an emerging biomarker for post-treatment surveillance in HPV-related head and neck squamous cell carcinoma, showing high accuracy for detecting recurrence. Studies have reported very high sensitivity and negative predictive value, with some demonstrating perfect predictive performance when ctHPV DNA is detected in serial plasma samples. Compared with conventional imaging such as PET-CT, ctHPV DNA may provide equal or superior diagnostic accuracy and can identify recurrence earlier. Overall, ctHPV DNA represents a promising non-invasive tool that complements imaging and improves post-therapy monitoring in HPV-associated HNSCC [22].

High-risk HPV genotype 16 is most strongly associated with the development of head and neck cancers. Prophylactic HPV vaccines protect against infection with high-risk HPV genotypes and have shown promising results in enhancing antitumor immune responses in HPV-positive OPSCC, particularly when combined with immune checkpoint inhibitors [23]. Currently, three HPV vaccination strategies are being investigated: postoperative administration to reduce the risk of recurrence, administration following chemoradiotherapy to prevent residual disease despite negative imaging findings, and preoperative administration to enhance the antitumor immune response [23].

The main HPV-related biomarkers discussed in this section, together with their clinical applications and current validation status, are summarized in Table 1.

#### 3.1.2. DNA Methylation

In HPV-positive oropharyngeal squamous cell carcinoma, 20 distinct differentially methylated regions have been identified across several genes and have been proposed as possible diagnostic markers for individuals with HPV-positive OPSCC: ATP5F1EP2, BEND4, C1QL3, CCDC181, CDKN2A, CTNND2, DPP4, ELMO1, HOXB4, ITGA4, KCNA3, NID2, OR6S1, SFMBT2, VSTM2B, ZNF137P, ZNF439, ZNF773, and ZNF93 [24]. Among the zinc finger gene family members, differential methylation of ZNF14, ZNF160, and ZNF420 has been identified as a highly specific biomarker for HNSCC detection, demonstrating 100% specificity in both primary tissue and saliva samples. In addition, hypermethylation of ZNF582 associated with PAX1 exhibits distinct methylation patterns in the oral cavities of patients with oral squamous cell carcinoma. Detection of these epigenetic alterations in exfoliated oral epithelial cells has shown potential utility in the diagnosis of OSCC [25].

Liouta et al. conducted a comprehensive review study integrating gene alterations induced by both hypomethylation and hypermethylation, evaluating their utility in the diagnosis, prognosis, and treatment response of HNSCC [24]. Hypermethylation of the DCC (a tumor suppressor gene that encodes a transmembrane protein involved in epithelial and neuronal cell differentiation) and EDNRB (encodes the endothelin B receptor) genes has been associated with malignant histopathological findings, independently of major risk factors such as age, tobacco use, and alcohol consumption. The association between the altered methylation status of these genes and malignant transformation suggests their potential utility as individual biomarkers for identifying patients at increased risk of developing head and neck cancer, particularly oral cancer, according to Gonzalez et al. [24,26]. Methylation alterations involving the 5′ and 3′ regions of the mediator complex subunit 15 gene (MED15) have been identified in HNSCC tumor samples obtained from smokers. These findings suggest a potential association between tobacco exposure, MED15 methylation abnormalities, and tumor development in HNSCC [24]. Hypermethylation has been observed in genes such as HOXA9, PROM1, OPRL1, and OPRM1, with higher methylation levels in tumor tissue compared to normal controls. These changes are suggested to have potential diagnostic value, particularly for early detection. These changes were also associated with an increased risk of recurrence [24]. In contrast, hypomethylation has been reported in genes such as PLAU, MTHFD1L, and XPR1 and has been associated with increased gene expression, potentially contributing to tumor progression and metastatic disease [24].

Hypermethylation of genes such as PDCD1, GALR2, SSTR1, SST, COL1A2, DAPK1, TAC1, GALR1, NPY1R, PITX1, C5orf66-AS1, RYR2 and PROM1 has been linked to low overall survival, higher recurrence risk, and advanced tumor stages, with additional associations having been reported with HPV status, smoking, and lymph node metastasis. Increased methylation of HOXA9 and altered methylation patterns of XPR1 have been associated with tumor progression, metastatic potential, and advanced clinical stage. In contrast, hypomethylation or increased expression of genes such as NID2, RIPOR3, CTLA4, and TNFSF11 has been associated with improved survival outcomes [24].

Recent studies on HNSCC have focused on identifying genes whose differential methylation patterns are associated with resistance to anti-EGFR therapies. Aberrant methylation of ErbB1/EGFR is associated with resistance to chemoradiotherapy and has been correlated with advanced-stage disease, poorly differentiated tumors, and metastatic spread [27]. In this context, hypermethylation and consequent transcriptional silencing of the death-associated protein kinase gene (DAPK) have been associated with reduced responsiveness to anti-EGFR agents. Accordingly, DAPK has been proposed as a predictive biomarker of resistance to anti-EGFR therapy and as a potential therapeutic target for overcoming treatment resistance [24]. Promoter methylation of the CCND2 oncogene has been linked to radiation resistance, while overexpression of CD47 has been identified as a characteristic feature of radioresistant tumor phenotypes. In addition, hypermethylation of the CCND1 oncogene, which encodes cyclin D1, has been associated with disruption of cell-cycle regulation and treatment resistance [24].

In the context of chemotherapy, some genes, including CRIP1, G0S2, MLH1, OPN3, the S100 calcium-binding family, and TUBB2A, have been reported to exhibit hypermethylation in cisplatin-resistant tumors, suggesting their involvement in treatment resistance mechanisms [24].

Regarding immunotherapy, methylation of the CD274 (PD-L1) promoter has been associated with modulation of the PD-1/PD-L1 axis and an inverse relationship with CD8+ T-cell infiltration, indicating its potential utility in predicting response to immune checkpoint blockade. Furthermore, altered methylation and expression patterns of GITR and OX40 have been associated with differential immunological activity and have been proposed as potential biomarkers for identifying patients who may benefit from adjuvant immunotherapy in HNSCC [24].

In laryngeal squamous cell carcinoma (LSCC), the most frequently altered gene is tumor protein 53. This gene encodes the p53 protein, which plays a central role in antitumor defense and is widely referred to in the literature as the “guardian of the genome”. Methylation of this gene leads to suppression of p53 production, resulting in uncontrolled cellular proliferation [27]. Furthermore, hypermethylation of the MGMT (O6-methylguanine-DNA methyltransferase) gene has well-established clinical significance. MGMT encodes a DNA repair enzyme responsible for preserving genomic integrity, including that of tumor cells. When the gene is methylated, laryngeal cancer cells become more sensitive to the effects of chemotherapeutic agents [27].

A summary of the principal DNA methylation biomarkers, including their associated HNSCC subsite, specimen, clinical application, validation status, and supporting references, is presented in Table 2.

#### 3.1.3. microRNAs

Tumor-associated microRNAs (miRNAs) are small non-coding RNA molecules involved in the regulation of gene expression as well as in intercellular communication. They are found in cellular environments and also in extracellular ones (bodily fluids such as blood, saliva, etc.). In extracellular environments, they can be identified as circulating microRNAs [28].

Through extensive studies, dozens of microRNAs have been identified as being involved in tumor development, prognosis, and treatment response in HNC. Due to the large volume of available data, we chose to focus this section on microRNAs that have been most extensively investigated and have shown promising diagnostic, prognostic, or predictive potential. Nevertheless, it should be noted that the literature contains numerous ongoing studies investigating additional RNA molecules that may further expand the biomarker landscape in head and neck oncology. Accumulating evidence suggests an association between miR-21 and cancers of the tongue, oral cavity and larynx, primarily through the inhibition of tumor cell apoptosis. This effect is mediated by the downregulation of PTEN (Phosphatase and Tensin Homolog)**,** a tumor suppressor gene, as well as PDCD4 (Programmed Cell Death 4)**,** a gene involved in the regulation of tumor invasion and metastasis. Furthermore, increased miR-21 expression has been associated with the progression of oral leukoplakia to squamous cell carcinoma and a poor prognosis in laryngeal cancer [29,30]. Overexpression of miR-31 has been associated with advanced tumor stages in OSCC and chemotherapy resistance [31]. Circulating levels of miR-31 in both saliva and plasma have been shown to correlate with tumor burden. Consequently, surgical resection of oral tumors has been associated with a significant reduction in salivary and plasma miR-31 levels, supporting its potential role as a biomarker for disease monitoring [30]. Patel et al. reported that increased expression of miR-1307-5p and reduced expression of miR-140, miR-143, and miR-145 was associated with resistance to chemotherapy. These findings suggest that alterations in the expression profiles of these microRNAs may serve as useful biomarkers for monitoring therapeutic response and treatment effectiveness in patients with head and neck cancer [32,33].

We also identified a comprehensive study addressing chemoresistance in relation to different classes of chemotherapeutic agents. Tumor resistance to Cetuximab was associated with the overexpression of miR-15b-5p, miR-92a, miR-548b, miR-103, miR-18a, miR-205, miR-532, miR-20a, and miR-365, whereas resistance to Cisplatin, Doxorubicin, and Fluorouracil was associated with increased expression of miR-494 [34]. The promoter methylation status of microRNAs like miR-9-1, miR-9-2, and miR-9-3 has been investigated in patients with HNSCC. Studies have shown that oral and oropharyngeal tumors tend to present higher methylation levels of miR-9-1 and miR-9-3 compared with laryngeal carcinomas, which is associated with decreased miR expression.

The diagnostic utility of salivary microRNAs in HNSCC may be influenced by several environmental and lifestyle factors, including diet, oral hygiene, alcohol consumption, smoking, and age-related changes. These variables can modify salivary miRNA expression, potentially affecting biomarker reliability. For normalization purposes, miR-16-5p has been identified as a stable and consistently abundant reference microRNA in healthy individuals [35].

Based on the available literature, we compiled a table summarizing the most extensively studied microRNAs in head and neck cancers. However, it should be noted that numerous additional microRNAs are currently under investigation and may further expand the range of clinically relevant biomarkers in the future (Table 3).

### 3.2. Proteomics

#### 3.2.1. EGFR (Epidermal Growth Factor Receptor)

Epidermal growth factor receptor (EGFR), also known as HER1 or ErbB1, is a transmembrane protein with receptor tyrosine kinase activity that belongs to the ErbB family. Binding of its ligands triggers the activation of one of three major signaling pathways described in the literature: the RAS–RAF–MAPK pathway, which promotes cellular proliferation and invasion; the PI3K/AKT pathway, which inhibits apoptosis; and the JAK/STAT pathway, which regulates gene transcription and supports cell survival. Overexpression of EGFR has been associated with poor prognosis in HNSCC and represents one of the clinically validated biomarkers currently used in therapeutic decision-making. As a result, EGFR has become an important target for modern anticancer therapies, despite being frequently overexpressed in HNSCC tumors [36]. Agents targeting EGFR belong to two major classes: monoclonal antibodies and tyrosine kinase inhibitors. The former includes Cetuximab, the first monoclonal antibody approved by the FDA for the treatment of HNSCC. Tyrosine kinase inhibitors are classified into three generations, the latest of which includes Osimertinib, a drug currently being investigated for its potential to target molecular mechanisms associated with resistance to chemoradiotherapy.

EGFR overexpression has been correlated with advanced tumor stage, poor histological differentiation, and metastatic disease, whereas lower EGFR expression has been associated with a more favorable response to neoadjuvant chemotherapy, radiotherapy, and organ-preserving surgical approaches. Currently, EGFR-targeted therapies remain an active area of research and development, with ongoing efforts aimed at improving treatment efficacy and overcoming therapeutic resistance in HNSCC.

#### 3.2.2. p53

The tumor suppressor protein gene TP53 encodes the p53 protein, which plays a critical role in the regulation of cell division and maintenance of genomic stability. Mutations in TP53 may lead to the emergence of mutant p53 proteins that promote uncontrolled cellular proliferation, tumor invasion, and metastasis, while also being associated with poor overall survival and resistance to chemoradiotherapy. Furthermore, TP53 alterations have been linked to genomic instability and a high tumor mutational burden [37]. TP53 mutations are frequently encountered in HNSCC, particularly in HPV-negative tumors. In some cases, these alterations may occur as part of Li-Fraumeni syndrome, a hereditary cancer predisposition syndrome characterized by the development of multiple primary malignancies. Kang et al. reported that treatment with immune checkpoint inhibitors is unlikely to significantly improve survival in tumors harboring TP53 mutations in the absence of additional actionable genetic alterations [37].

#### 3.2.3. VEGFA (Vascular Endothelial Growth Factor-A)

VEGFA, a growth factor involved in tissue repair and angiogenesis, regulates the formation of new blood vessels and consequently increases the supply of oxygen and nutrients to tissues. In HNSCC, alterations affecting VEGFA, most commonly promoter hypomethylation, have been associated with enhanced angiogenesis and tumor growth [38]. Due to its role in tumor progression, VEGFA has been investigated as a potential diagnostic and prognostic biomarker in head and neck cancers. In addition, several promising studies have explored the potential role of Simvastatin, a cholesterol-lowering medication, in reducing the expression of both VEGFA and EGFR, suggesting a possible adjunctive therapeutic application in HNSCC [38].

#### 3.2.4. Transforming Growth Factor (TGF)

Transforming growth factor is a cytokine involved in the regulation of the cell cycle and tissue homeostasis. During the early stages of tumorigenesis, it acts as a tumor suppressor by inhibiting cellular proliferation. However, in advanced stages of cancer, TGF contributes to the maintenance of the tumor microenvironment by promoting inflammation, tumor cell proliferation, and disease progression [2].

#### 3.2.5. Fibroblast Growth Factor Receptor 1 (FGFR1)

FGFR1 is overexpressed in approximately 20% of squamous cell carcinomas and has been associated with reduced overall survival. Due to its role in tumor progression, FGFR1 has emerged as a potential therapeutic target, with FGFR inhibitors being investigated for use in tumors exhibiting increased FGFR1 expression [39].

**Table 3:** Types of microRNAs and their clinical applications in HNSCC.

HNSCC Subsite	MiRNA Type	Clinical Use	Study
Oral cavity, tongue	miRNA (miR-21, miR-145, miR-93, miR184, miR-31, miR-412-3p, miR-34a) (upregulation) miRNA (miR27b, miR-200, miR375, miR-26a, miR-7, miR-107, miR-218, let-7, miR-125) (upregulation) miRNA (miR-24, miR-3p, miR-10b-5p, miR-134, miR-200, miR-302b-3p, miR-365, miR-486-5p, miR-512-3p, miR-4484) (upregulation)	progression of oral leukoplakia, invasion, metastasis diagnosis, prognosis (tumor suppressors) early diagnosis, poor prognosis	Osan et al. [30,31] Manzano-Moreno et al. [40] Dioguardi et al. [31] Manzano-Moreno et al. [40] Manzano-Moreno et al. [40]
Larynx	miRNA-21, miRNA-146 (upregulation) miRNA-20b (downregulation) miRNA-766-5p (downregulation) miRNA-23a (upregulation) miRNA26a/26b (upregulation) miRNA-31, miRNA-196b (upregulation) miRNA-34s (a, b and c) (downregulation) miRNA-144-3p, miRNA-126, miRNA-181a, miRNA-203, miRNA-195, miRNA-519a (downregulation) miRNA-144b, miRNA-93 (upregulation) miRNA-9 (upregulation) miRNA-101, miRNA-200, miRNA-29c (downregulation) miRNA-296-5p (upregulation) miRNA-155 (upregulation)	poor prognosis disease-free survival and overall survival poor overall survival tumor progression, invasion poor overall survival metastasis, high-grade differentiation high risk of recurrence tumor suppressor, good prognosis poor prognosis invasion, metastasis low overall survival, metastasis, recurrence, heavy smoking recurrence, radioresistance advanced stage	Komitova et al. [29] Komitova et al. [29] Komitova et al. [29] Komitova et al. [29] Komitova et al. [29] Komitova et al. [29] Komitova et al. [29] Komitova et al. [29] Komitova et al. [29] Komitova et al. [29] Komitova et al. [29] Komitova et al. [29] Komitova et al. [29]
Oropharynx	miRNA-9-1 miRNA-9-2 miRNA-9-3 (methylation)	diagnosis of OPSCC	Liouta et al. [24]

#### 3.2.6. Mesenchymal–Epithelial Transition Factor (c-MET)

c-MET is a membrane tyrosine kinase receptor activated primarily by its ligand, the hepatocyte growth factor (HGF). Aberrant activation of the HGF/c-MET signaling pathway has been associated with enhanced tumor proliferation, invasion, angiogenesis, and metastatic potential, contributing to the development and progression of head and neck cancers. The tumorigenic functions of c-MET are documented to be associated with the differentiation of regulatory T cells and their accumulation in tumors, thus promoting tumor growth and metastasis [41]. In addition, c-MET dysregulation has been linked to treatment resistance, making this pathway a target for the development of novel targeted therapies in HNSCC [41].

#### 3.2.7. Heat Shock Proteins (HSP)

Heat shock proteins are intracellular chaperone proteins induced by oxidative stress, hypoxia, ethanol exposure, infections, and radiation. They play a role in maintaining cellular homeostasis by preventing inappropriate protein folding, thereby exerting cytoprotective effects. However, when present in excessive amounts, they may become pathogenic, contributing to uncontrolled cellular proliferation and mechanisms of resistance to anticancer therapies [42].

Phosphorylation of HSP27 in LSCC has been associated with resistance to platinum-based chemotherapy, uncontrolled cellular proliferation, and metastasis. In contrast, overexpression of HSP70 has been linked to radioresistance through the inhibition of radiation-induced ROS-mediated apoptosis. HSP47 overexpression has been associated with poor prognosis in OSCC; however, paradoxically, in LSCC it has been correlated with increased sensitivity to cisplatin and improved overall survival [42].

#### 3.2.8. Metallothioneins

Metallothioneins are metal-binding proteins that protect cells against reactive oxygen species, radiation, and heavy metals. Under physiological conditions, metallothioneins act as tumor suppressors; however, when overexpressed, they may inhibit p53 function, resulting in uncontrolled cellular proliferation. Data from the literature have correlated metallothionein overexpression with a potential predictive role in the malignant transformation of benign lesions in LSCC [42].

#### 3.2.9. Heme Oxygenase and Nuclear Factor Erythroid 2-Related Factor 2 (Nrf2)

Heme oxygenase is an enzyme that protects cells, including tumor cells, from apoptosis. Cellular oxidative stress activates Nrf2, which binds to the heme oxygenase gene and promotes its expression. In HNSCC, heme oxygenase is frequently overexpressed, thereby reducing the pro-apoptotic effects of Cisplatin and radiotherapy and contributing to treatment resistance. This may also result in the need for higher Cisplatin doses, leading to increased systemic toxicity. We found some articles suggesting that suppression of heme oxygenase expression through molecular approaches could enhance Cisplatin chemosensitivity, potentially allowing the use of lower therapeutic doses [42].

#### 3.2.10. Cyclooxygenase-2 (COX-2)

Cyclooxygenase-2 (COX-2) is an inducible enzyme expressed under conditions of cellular stress, including radiotherapy and tobacco exposure. It catalyzes the conversion of arachidonic acid into prostaglandins, particularly prostaglandin E2 (PGE2), which plays a key role in inflammation and tumor progression. Elevated COX-2 levels have been associated with an increased risk of local recurrence following surgical treatment, even in early-stage disease, as well as a higher risk of metastasis and radioresistance. Several studies are currently investigating the therapeutic potential of COX-2 inhibition. Celecoxib, a selective COX-2 inhibitor, has been shown to enhance the immune response by promoting natural killer (NK) cell proliferation, thereby potentially improving the efficacy of immunotherapy. In addition, in vitro inhibition of COX-2 has been associated with increased sensitivity to taxanes, while selective inhibitors such as NS-398 and SC-236 may enhance tumor radiosensitivity [42].

#### 3.2.11. Cytoskeletal and Cell Membrane Proteins

Integrins are transmembrane proteins involved in cell adhesion, differentiation, and proliferation. They consist of α and β subunits that combine to form heterodimers. Altered integrin expression has been associated with tumor invasion and metastatic spread. Several studies have investigated the role of integrin αVβ6 in oral squamous cell carcinoma, demonstrating a strong association with a poor prognosis and reduced overall survival. Sakurai et al. reported upregulation of focal adhesion kinase (FAK), a key molecule involved in signal transduction, in OSCC and highlighted its potential utility as a prognostic factor for overall survival. The same study also identified integrin β8 as a predictive biomarker of lymph node metastasis in OSCC [43].

E-cadherin is a transmembrane glycoprotein involved in cell adhesion, while cyclin D1 is involved in cell division, with both being used as tumor biomarkers in some studies. Dar et al. conducted a study demonstrating that cyclin D1 overexpression and E-cadherin downregulation are early alterations that occur during the carcinogenesis of OSCC [44,45].

Podoplanin is a mucoprotein expressed by lymphatic endothelial cells. Its overexpression has been associated with tumor progression and lymph node metastasis [2].

Dipeptidyl peptidase-4 (DPP-4) is a cell surface glycoprotein involved in glucose metabolism and immune regulation. Tarrad et al. investigated the diagnostic value of both salivary and plasma DPP-4 levels, demonstrating that salivary DPP-4 showed a stronger correlation with tumor grade in OSCC than its plasma counterpart [46].

Elevated cytokeratin 17 (also known as K17 or stress keratin) expression at either the RNA or protein level has been associated with an unfavorable prognosis [47]. K17 is a type I intermediate filament protein that is typically expressed during embryonic development and is transiently induced following tissue injury. In normal adult tissues, its expression is largely silenced, with the exception of certain stem cell compartments; however, it is re-expressed in multiple malignancies. Emerging evidence suggests that K17 contributes to tumorigenesis through diverse mechanisms, including modulation of transcriptional programs, regulation of subcellular protein localization, metabolic reprogramming such as enhanced glycolysis, and promotion of cancer stem cell-like properties and immune evasion. Nevertheless, the precise molecular pathways through which K17 influences tumor behavior and patient prognosis remain incompletely understood and require further investigation [47,48].

Stathmin (Oncoprotein 18) is a cytoskeletal protein that is overexpressed in OSCC. Its increased expression has been associated with tumor invasion, metastasis and, consequently, more advanced TNM stages, as well as a poorer long-term prognosis [49].

Claudin-1 is a transmembrane protein with an emerging role as a therapeutic biomarker. It is involved in myoepithelial transformation and fibrogenesis and has been associated with the development of conditions characterized by extensive fibrosis. The highest levels of Claudin-1 expression have been reported in HPV-positive tumors [50].

#### 3.2.12. Aldehyde Dehydrogenase

Aldehyde dehydrogenases (ALDHs) represent a family of enzymes comprising 19 active isoforms involved in cellular detoxification, protection against oxidative stress, and retinoic acid synthesis through the catabolism of aldehydes into carboxylic compounds [51]. In cancer cells, ALDH enzymes are overexpressed and contribute to cellular proliferation, differentiation, and poorer overall survival by reducing the formation of reactive oxygen species and generating retinoic acid, thereby exerting a significant impact on the tumor microenvironment. A small proportion of tumor cells exhibit stem cell-like characteristics and are involved in tumor proliferation, metastasis, and treatment resistance. Among the biomarkers associated with cancer stem cells, CD44/ALDH1A1 are the most extensively studied [27].

Many anticancer therapies rely on the generation of aldehydes and reactive oxygen species, whose cytotoxic effects induce tumor DNA damage and apoptosis of malignant cells. Cytosolic ALDH1 isoforms participate in the conversion of vitamin A into retinal and subsequently into retinoic acid, which binds to retinoic acid receptors in the nucleus. This signaling pathway has been associated with treatment resistance and poorer overall survival through inhibition of apoptosis mediated by the activation of cyclin D1 and c-MYC [51].

Multiple ALDH inhibitors are currently being investigated as potential strategies to overcome resistance to chemotherapy. The association of Disulfiram (an ALDH inhibitor) with Cisplatin increased treatment response in LSCC [27]. A theophylline-based molecule has demonstrated promising in vitro activity in suppressing tumor growth. Furthermore, the ALDH inhibitor Aldi-6, when combined with Cisplatin, has been shown to reduce tumor growth in vitro, while Dyclonine in combination with Sulfasalazine has been reported to suppress the growth of tumors expressing the ALDH3A1 isoform [51,52].

#### 3.2.13. Fibrinogen

Plasma fibrinogen levels have been found to be elevated in OSCC and may serve as a potential tumor biomarker due to their increased expression in affected patients. In addition, fibrinogen has been implicated in angiogenesis, further supporting its role in tumor progression [53].

#### 3.2.14. Ki-67

Ki-67 is a protein used as a marker of tumor aggressiveness, closely associated with uncontrolled cellular proliferation and tumor progression. Increased Ki-67 expression has been linked to a higher risk of lymph node metastasis and more advanced locoregional tumor stages. In contrast, lower Ki-67 levels have been associated with slower tumor growth and reduced biological aggressiveness [49].

#### 3.2.15. Cathepsin B

Cathepsin B is an enzyme belonging to the protease family that plays a role in tumor invasiveness and metastasis. Studies have shown that elevated salivary Cathepsin B levels are associated with high sensitivity and specificity for the diagnosis of OSCC. Its increased expression has also been correlated with tumor volume, supporting its value as a diagnostic biomarker [49].

#### 3.2.16. Survivin

Survivin is a protein that blocks apoptosis. Overexpression of survivin has been associated with exposure to established risk factors, including alcohol consumption, tobacco use, and betel nut chewing. Poorly differentiated tumors have been shown to exhibit higher Survivin expression levels, which have been linked to resistance to conventional oncologic treatments [49].

#### 3.2.17. Cornulin

Cornulin is a protein specific to squamous epithelium and is predominantly expressed in the superficial epithelial layers. It is considered a stress-responsive protein, as its upregulation has been associated with reduced apoptosis. Cornulin plays an important role in epidermal differentiation, and its expression gradually decreases as tumors progress. Studies have demonstrated that salivary Cornulin exhibits high sensitivity and specificity as a screening biomarker for OSCC. Furthermore, elevated Cornulin and Keratin 4 expression at the surgical margins of resected tumors has been associated with an increased risk of tumor recurrence [54].

#### 3.2.18. Parkinson’s Disease Protein 7 (PARK7/DJ-1)

Parkinson’s disease protein 7 (PARK7/DJ-1) is a protein that is overexpressed in multiple types of cancer and has been implicated in carcinogenesis, metastatic progression, and reduced overall survival. PARK7 is particularly overexpressed in OSCC, where it has emerged as a promising biomarker for the early detection of oral cavity and tongue cancers [55].

#### 3.2.19. Matrix Metalloproteinase (MMP) and Platelets

MMP-9 and MMP-2 expression is induced by platelets, which protect metastatic tumor cells from immune surveillance by forming a protective matrix around them. This process facilitates the release of growth factors from the extracellular matrix. In addition, platelets promote increased vascular permeability and support the extravasation of tumor cells into the bloodstream, thereby contributing to metastatic dissemination [56].

#### 3.2.20. Biomarkers of Bone Resorption

N-telopeptide of type I collagen (NTx) and C-telopeptide (CTx) are considered biomarkers of bone resorption that reflect osteoclastic activity and increased bone turnover. In a study conducted by Singh et al., a considerable proportion of patients with OSCC exhibited elevated serum NTx and CTx levels despite the absence of radiologically detectable bone invasion. Furthermore, increased NTx concentrations were associated with poorer clinical outcomes, suggesting a potential role for this biomarker in predicting disease progression [57].

#### 3.2.21. Hypoxia-Inducible Factor

Cellular hypoxia may, under the influence of various genetic, environmental, and lifestyle-related factors, trigger adaptive mechanisms that allow cells to survive rather than undergo apoptosis. One of the most extensively studied mediators of the hypoxic response is hypoxia-inducible factor-1 alpha (HIF-1α). Other molecules involved in this process include glucose transporter-1 (GLUT-1) and carbonic anhydrase IX (CA-IX). HIF-1α expression has been reported to be more pronounced in highly keratinized regions of tumors. Both HIF-1α and HIF-2α are currently being investigated as potential therapeutic targets in HNSCC [58,59].

#### 3.2.22. Perforin-1

Perforin-1 is a protein secreted by cytotoxic T lymphocytes and NK cells and plays an important role in cell-mediated immunity. Under physiological conditions, it contributes to antitumor defense by inducing apoptosis of malignant cells, thereby limiting tumor growth and metastatic spread. Reduced Perforin-1 expression has been associated with enhanced tumor proliferation and metastasis, likely due to impaired recognition and depletion of transformed cells by the immune system [60].

The main proteomic biomarkers discussed in this section, together with their categories, clinical applications, current clinical status, and supporting references, are presented in Table 4.

### 3.3. Cytokines

IL-6 is a predictive biomarker of recurrence and prognosis in HNSCC and is closely associated with extensive inflammatory activity. Lower serum IL-6 levels have been correlated with a more favorable response to Cetuximab therapy [61].

Several chemokines and their receptors have been implicated in the progression and metastatic behavior of OSCC. CXCL1 has been associated with reduced survival outcomes [61]. Increased expression of CCL2 has been associated with poorly differentiated tumors, advanced disease stages, and metastatic potential. By comparison, CCL3 and CCL4 have not demonstrated a strong association with carcinogenesis in previous studies. Likewise, overexpression of CXCR4 has consistently been linked to tumor progression, lymph node metastasis, and reduced survival, supporting its value as a prognostic biomarker. Elevated CXCR2 expression has also been correlated with nodal involvement, while the CXCR3/CXCL9 axis has been associated with cervical lymph node metastasis and poorer outcomes in OSCC.

IL-10 levels have been found to be significantly increased in tumor tissues from patients with HPV-positive OSCC compared with healthy tissues. Elevated salivary IL-10 concentrations have also been reported and have been associated with poorer clinical outcomes. In addition, increased IL-17 levels have been detected in advanced stages of OSCC, where they have been linked to lymphangiogenesis and disease progression [62].

Although the exact role of XCR1 remains unclear, current evidence suggests a possible involvement in OSCC metastasis [63,64].

The cytokine and chemokine biomarkers discussed in this section are summarized in Table 5.

### 3.4. Tumor Microenvironment (TME)

The tumor microenvironment is involved in carcinogenesis, immune suppression, poor treatment response, and unfavorable long-term outcomes in cancer, with the degree of tumor immunogenicity being closely related to prognosis. Among the biomarkers involved in the characterization of the tumor microenvironment are immune checkpoint molecules, microsatellite instability (MSI), tumor mutational burden (TMB), and tumor-infiltrating lymphocytes (TILs) [27].

Immune checkpoints include both co-stimulatory molecules (CD27, CD28, CD40, and CD137/4-1BB) and co-inhibitory molecules (PD-1, PD-L1, and CTLA-4), which regulate T-cell activity. The most extensively studied immune checkpoint inhibitors target PD-1, PD-L1, and CTLA-4. PD-1 (CD279) is expressed on a variety of immune cells and plays a role in suppressing the activity of tumor-infiltrating T lymphocytes. It interacts with two ligands, PD-L1 (B7-H1/CD274) and PD-L2 (B7-DC/CD273), the former being more frequently expressed by tumor cells. In addition to its expression on tumor cells, PD-L1 can also be detected in peripheral blood in several forms, including as a soluble protein, within extracellular vesicles, and on circulating tumor cells. This expands the potential applications of PD-L1 testing by enabling liquid biopsy approaches. Elevated circulating PD-L1 levels have been associated with unfavorable clinical outcomes and poorer prognosis in patients with HNSCC [65].

CTLA-4 (CD152) is expressed by activated T cells (CD4+ and CD8+) as well as regulatory T cells [66].

Recent evidence suggests that circulating CD3+CD137+ lymphocytes may serve as a predictive biomarker of response to ICIs in patients with recurrent or metastatic HNSCC, with higher pre-treatment levels having been associated with a more favorable response to immunotherapy [65]. Additionally, an elevated neutrophil-to-lymphocyte ratio has consistently been linked to reduced survival in patients with HNSCC [67].

Tertiary lymphoid structures are ectopic lymphoid aggregates that develop within non-lymphoid tissues in response to chronic inflammatory stimuli, including cancer. Structurally, they contain B-cell follicles and T-cell zones with high endothelial venules that facilitate local antigen presentation. In HNSCC, tumors with tertiary lymphoid structures are characterized by abundant B- and T-cell infiltration and have been associated with a more favorable prognosis and improved responsiveness to immunotherapy. Similarly, increased infiltration by immune cells within the tumor microenvironment, particularly CD56+ natural killer cells, has been correlated with enhanced responses to ICI therapy [67].

Among PD-1/PD-L1-targeted therapies, Pembrolizumab and Nivolumab have been approved by both the FDA and EMA for the treatment of advanced, unresectable, recurrent, or metastatic head and neck cancers [68]. Durvalumab and Atezolizumab are still under investigation, while Cemiplimab has demonstrated promising results in cutaneous squamous cell carcinomas. PD-L1 expression is currently used as a predictive biomarker for response to PD-1 inhibitors and is clinically assessed using the Combined Positive Score (CPS), with threshold values of ≥20 and ≥1 considered clinically significant [67].

Despite these advances, studies have shown that only a minority of patients (approximately 20%) achieve significant clinical benefit from immunotherapy alone, highlighting the need for combination strategies involving radiotherapy and chemotherapy. An important consideration is the higher immunogenicity of HPV-positive tumors compared with HPV-negative tumors, which may partially explain their improved response to immune-based therapies [67]. Tobacco exposure also affects the TME, particularly in HPV-negative cancers. Patients with a history of smoking have been shown to exhibit a poorer response to immunotherapy compared with non-smokers [2].

Additionally, the accumulation of immunosuppressive cell populations, including myeloid-derived suppressor cells, M2-polarized tumor-associated macrophages, and N2 tumor-associated neutrophils, has been correlated with poorer responses to ICI treatment [65]. Expression of CD73 has also been associated with reduced responsiveness to immunotherapy [65].

An emerging area of interest in the field of biomarkers is the evaluation of type I and type II interferons expressed by natural killer cells. These cytokines have been shown to induce PD-L1 overexpression [65].

Another emerging immune checkpoint target is lymphocyte activation gene-3 (LAG-3). Eftilagimod alpha, a therapeutic agent targeting this pathway, has been shown to act synergistically with PD-L1 inhibitors by enhancing immune cell infiltration within the tumor microenvironment. Within the tumor-infiltrating lymphocyte population, overexpression of T-cell immunoglobulin and mucin-domain-containing-3 (TIM-3) has been associated with advanced locoregional disease and lymph node metastasis. Multiple studies evaluating anti-TIM-3 monoclonal therapies are currently underway [67].

Tumor mutational burden (TMB) represents the total number of mutations present within a tumor genome. Tumors characterized by a high TMB are generally considered more immunogenic, and a value of ≥10 mutations/Mb is commonly regarded as elevated and potentially predictive of benefit from immunotherapy. However, considerable variability exists across studies regarding the optimal cutoff value, with reported thresholds ranging from 6 to 175 mutations per exome. Microsatellite instability (MSI) is characterized by deficiencies in the DNA mismatch repair (MMR) system. Reduced expression of mismatch repair proteins has been associated with increasing degrees of epithelial dysplasia. In addition, several studies have suggested a role for MMR system alterations in the development of resistance to platinum-based chemotherapy [69]. Nevertheless, the prevalence of MSI in HNSCC is relatively low, and at present, neither the FDA nor the EMA routinely recommends MSI testing in this tumor type [65,67].

An overview of the principal tumor microenvironment biomarkers is presented in Table 6.

### 3.5. Metabolomics

Metabolic reprogramming is a hallmark of HNSCC, contributing to tumor initiation, progression, immune evasion, and treatment resistance. Consequently, several metabolites and metabolism-related enzymes have emerged as promising biomarkers.

#### 3.5.1. Tryptophan Metabolism

Indoleamine 2,3-dioxygenase (IDO) is an enzyme involved in the metabolism of L-tryptophan and the production of immunoregulatory metabolites, thereby limiting excessive inflammation and T-cell activation. It has emerged as a predictive biomarker of response to immunotherapy, triggering immunotherapy resistance and a negative prognosis [2,65]. Kynurenine is a metabolite of the essential amino acid L-tryptophan, generated through its catabolism by tryptophan dioxygenase and indoleamine 2,3-dioxygenase. It contributes to cellular energy production through the synthesis of NAD+ and has also been implicated in tumor angiogenesis [70].

#### 3.5.2. Glycosylation Biomarkers

Sialic acid is a glycoconjugate molecule located on the cell membrane and is involved in cell adhesion and intercellular interactions. Aberrant glycosylation is a recognized feature of oral cavity and tongue cancers, and sialic acid-containing glycoconjugates have been proposed as useful biomarkers of malignant transformation. Increased sialylation and enhanced sialyltransferase activity have been associated with greater tumor invasiveness and metastatic potential. By modifying the tumor cell surface, these alterations facilitate immune evasion, promote cellular adhesion, and support the dissemination of malignant cells, thereby contributing to tumor progression [71].

#### 3.5.3. Oxidative Stress Biomarkers

Repeated exposure of the mucosal tissues to carcinogens, such as tobacco smoke, induces the generation of reactive oxygen species. Their excessive accumulation, together with a decline in antioxidant defenses, leads to the development of oxidative stress. Free radicals damage cellular membranes and promote lipid peroxidation, resulting in the formation of lipid hydroperoxides and malondialdehyde. The latter has emerged as a promising biomarker for the diagnosis and management of HNSCC [72].

Oxidative stress has been associated with uncontrolled cellular proliferation. In a study conducted by Drotárová et al., reduced superoxide dismutase (SOD) activity was found to be correlated with carcinogenesis in OSCC. In addition, as part of an adaptive response to increased oxidative stress, elevated levels of glutathione, glutathione reductase, and glutathione peroxidase were observed [73].

#### 3.5.4. Biomarkers of Cellular Energy Metabolism

Extracellular ATP contributes to tumor progression and chemotherapy resistance, with ATP levels being higher in cancer cells than in normal cells. Several studies are currently investigating therapeutic agents capable of inhibiting ATP synthesis, with the aim of enhancing tumor sensitivity to chemotherapy and improving treatment response [74].

Lactate dehydrogenase (LDH) is an enzyme that catalyzes the reversible conversion of lactate to pyruvate and NADH to NAD+ and exists in two major isoforms [8]. Several studies have shown that elevated serum LDH levels are associated with uncontrolled tumor cell proliferation, invasion, metastasis, and resistance to chemotherapy. LDH concentrations have been reported to increase progressively from the stage of potentially premalignant disorders to established malignancy [75]. Although LDH is not specific to HNSCC, as elevated levels are also observed in other malignancies such as breast and lung cancer, its overexpression has been associated with a poor prognosis and reduced overall survival [8,75].

Triosephosphate isomerase 1 (TPI1) is an enzyme involved in glucose metabolism and tumor cell activity, contributing to increased intracellular glucose availability and enhanced histone acetylation during the cell cycle. In a study published in 2023, Li et al. reported overexpression of TPI1 in LSCC, highlighting its potential role as a tumor biomarker. However, further studies are required to validate these findings and clarify their clinical significance [76].

The principal metabolomic biomarkers discussed in this section, including their metabolic pathways, proposed clinical applications, current clinical status, and supporting references, are presented in Table 7.

### 3.6. The Oral Microbiome as a Biomarker Source

The oral microbiome is increasingly recognized as a source of biomarkers in HNSCC, spanning from etiological risk to mechanistic drivers of pathogenesis, to predictors of treatment response. The oral microbiota comprises both a stable core community and inter-individual variability across healthy humans, with recognizable core taxa (Streptococcus, Prevotella, Veillonella, Neisseria, Rothia, Haemophilus, Porphyromonas) shaped by host factors [77]. This ecosystem supports oral homeostasis by protecting against pathogen colonization, modulating local immune responses, and contributing to nutrient metabolism [78]. However, factors such as diet, poor oral hygiene, and medication use may disrupt this balanced state, leading to lasting alterations in the oral ecosystem and promoting the transition from health to dysbiosis and disease [79]. Dysbiosis is often described as a targeted shift in certain microbial taxa rather than a global loss of diversity, but this model does not apply uniformly across oral diseases. In caries and periodontitis, dysbiosis has been characterized as a compositional shift among specific taxa within an already diverse ecosystem, occurring without significant loss of overall diversity [78,80]. In oral cancer, by contrast, dysbiosis follows a classical diversity-loss pattern, with patients showing significantly reduced alpha diversity and a distinct beta-diversity profile compared to healthy controls, alongside shifts in specific taxa such as reduced Corynebacterium and increased Dialister, Selenomonas, and Streptococcus [81]. In addition, reports revealed correlations between the abundances of some microbial species and cancer progression in HNSCC [81].

Oral dysbiosis is thought to contribute to carcinogenesis by activating oncogenic signaling such as Wnt/β-catenin while suppressing antitumor immune responses [2]. Its role also extends to broader immune regulation. Cattin et al. (2021) demonstrated that microbiota-derived signals shape immune responses through modulation of dendritic cell function and retinoic acid (RA) metabolism, notably by regulating the expression of aldehyde dehydrogenase (ALDH) enzymes, particularly ALDH1A2/RALDH2 [82]. These findings suggest a mechanistic link between microbial communities, immune regulation, and ALDH-dependent pathways. Of particular interest, ALDH activity is an intrinsic property of cancer stem cells [83]. However, the extent to which oral microbial dysbiosis influences ALDH activity, immune modulation, and cancer stem cell (CSC) maintenance in HNC remains largely unknown. Persistent inflammation driven by HPV infection has been associated with hypermethylation of the CDKN2A gene, which encodes the tumor suppressor protein p16INK4a, as well as increased EGFR expression. Among the microorganisms implicated in oral carcinogenesis, Fusobacterium nucleatum and *Porphyromonas gingivalis* have been associated with p53 suppression, oxidative stress, and DNA damage, while P. gingivalis has additionally been linked to local tumor invasion and chemotherapy resistance. Oral colonization by Fusobacterium nucleatum has also been reported more frequently in HPV-positive oral cavity tumors [84].

OSCC-associated dysbiosis has been characterized by an increased abundance of several bacterial species, including Peptostreptococcus stomatis, Haemophilus influenzae, Fusobacterium periodonticum (particularly in advanced disease), Parvimonas micra, Streptococcus constellatus, Filifactor alocis, Pseudomonas aeruginosa, and Veillonella spp. Treponema denticola, an anaerobic spirochete, promotes the expression of dentilisin, a protease implicated in tumor invasiveness. Advanced periodontal disease has also been associated with Aggregatibacter actinomycetemcomitans, a pro-inflammatory bacterium that promotes uncontrolled cellular proliferation and angiogenesis [84].

In addition, Streptococcus anginosus contributes to increased acetaldehyde production, leading to genomic instability and activation of inflammatory mediators such as IL-8, COX-2, and TNF-α. Candida albicans also participates in acetaldehyde production, and its coexistence with Fusobacterium nucleatum or *Porphyromonas gingivalis* has been associated with enhanced inflammation, reduced apoptosis, DNA damage, and uncontrolled tumor cell proliferation. Current research is increasingly focused on manipulating the oral microbiota as a potential adjuvant therapeutic strategy in the management of malignant diseases [84].

Several studies have associated recent antibiotic treatment, administered within approximately one month prior to the initiation of ICIs, with reduced treatment response and decreased survival [65]. Among the different classes of antibiotics, fluoroquinolones have been most strongly associated with a reduced response to ICI therapy. Therefore, in patients undergoing immunotherapy, unnecessary antibiotic use should be minimized whenever possible in order to preserve the local microbiome, which may play an important role in modulating treatment response [2].

The main microbiome-associated biomarkers discussed in this section, together with their proposed clinical applications, current clinical status, and supporting references, are presented in Table 8.

## 4. Discussion

### 4.1. Future of HNSCC Diagnosis and Treatment

HNSCC continues to show an increasing worldwide incidence, while overall survival remains relatively modest. Therefore, the identification of reliable biomarkers capable of stratifying patients, personalizing treatment, and predicting therapeutic response has become a major clinical priority. Although numerous tumor biomarkers have been investigated in recent years, the findings of this scoping review indicate that only a limited number of biomarkers have been translated into routine clinical practice, whereas most remain promising candidates requiring further clinical validation. Currently, HPV-related biomarkers (including p16INK4a) and PD-L1 assessed using the Combined Positive Score (CPS) for patient selection for immunotherapy represent the biomarkers with the strongest clinical applicability in HNSCC. Most genomic, proteomic, metabolomic, microbiome-associated, and tumor microenvironment biomarkers remain investigational despite encouraging results reported in the literature.

Genomic studies have provided valuable information regarding the relationship between gene alterations and carcinogenesis, while advances in tumor immunology have highlighted new directions for the development of targeted therapies. Likewise, proteomic research has identified several promising biomarkers with potential applications in diagnosis, prognosis, and personalized treatment. However, the available evidence remains heterogeneous because of differences in study design, investigated biomarkers, analytical methods, and reported clinical outcomes. Nevertheless, the molecular profile alone cannot fully explain tumor behavior, as each patient’s phenotype and biological response to environmental and lifestyle-related risk factors also contribute to disease development and treatment outcomes.

A multimodal approach combining several highly specific biomarkers according to the anatomical site of the squamous cell carcinoma, together with the development of integrated risk scores, may provide more accurate information for early diagnosis, prognostic assessment, and treatment selection. Continuous progress in molecular biology and high-throughput analytical techniques is expected to identify new therapeutic targets, ultimately improving overall survival, life expectancy, and quality of life. Furthermore, comprehensive molecular profiling may facilitate patient stratification by identifying those who are more likely to benefit from surgical treatment and those who may achieve better outcomes with radiotherapy, immunotherapy, or other oncological strategies. Future studies should focus on prospective validation of candidate biomarkers, standardization of analytical methodologies, and integration of multi-omics data to facilitate their translation into clinical practice.

Current research is also focused on identifying biomarkers capable of detecting premalignant lesions, representing another important step toward earlier diagnosis and cancer prevention. Despite these advances, several limitations still hinder the widespread implementation of biomarker-guided medicine, including the high cost of molecular testing, limited accessibility for routine large-scale screening, tumor heterogeneity, complex mechanisms of treatment resistance, and the remarkable molecular diversity of head and neck cancers. In addition, the absence of standardized testing protocols and universally accepted molecular techniques limits the routine use of many promising biomarkers. Consequently, clinicians still rely mainly on well-established biomarkers and conventional treatment strategies rather than individualized therapeutic approaches based on each patient’s molecular and phenotypic profile.

As molecular technologies continue to evolve, together with the rapid expansion of artificial intelligence, the field of tumor biomarkers is expected to develop considerably. These advances will likely accelerate the implementation of precision medicine, allowing treatment decisions to become increasingly tailored to the biological characteristics of each patient and each individual tumor. Nevertheless, the successful integration of novel biomarkers into routine clinical practice will depend on robust prospective validation, multicenter studies, and the establishment of standardized laboratory and reporting protocols.

### 4.2. Study Limitation

One limitation of the present review is the considerable methodological heterogeneity of the included studies, encompassing a broad range of tumor biomarkers, study designs, investigated populations, and reported outcomes. As this review was conducted as a scoping review, its primary objective was to comprehensively map the available evidence rather than to critically appraise individual studies or quantitatively synthesize the results. Therefore, the findings of the present review should be interpreted in consideration of the methodological heterogeneity of the available evidence. As evidence continues to evolve, future reviews focused on individual biomarkers or more homogeneous biomarker groups may facilitate systematic evidence synthesis and quantitative analyses where appropriate.

## 5. Conclusions

Continuous research has led to the identification of numerous biomarkers that may improve the diagnosis, prognosis, and treatment of head and neck cancers. Besides their potential role in early diagnosis, many of these biomarkers may help predict postoperative recurrence, treatment resistance, and long-term clinical outcomes. This scoping review highlights that, despite the growing number of candidate biomarkers, only a limited number are currently supported by sufficient evidence for routine clinical use, whereas most remain promising candidates requiring further prospective validation. Integrating genomic, proteomic, metabolic, tumor microenvironment, immune, and microbiota-related biomarkers may provide a more accurate characterization of tumor behavior than relying on a single biomarker. However, the translation of these biomarkers into routine clinical practice is currently limited by methodological heterogeneity, the lack of standardized analytical protocols, and the need for validation in large prospective multicenter studies. Further clinical validation and the development of standardized biomarker panels are needed before their widespread implementation in clinical practice, thereby paving the way toward precision medicine for patients with head and neck cancers.

## Figures and Tables

**Table 1:** HPV-related biomarkers in head and neck squamous cell carcinoma.

Biomarker	HNSCC Subsite	Detection Method/Specimen	Clinical Use	Current Clinical Status	Study
E6/E7 oncogene microRNA	Oropharynx	RT-PCR and ISH, tumor	Diagnostic, prognostic, predictive for treatment response	Recommended by NCCN in OPSCC	Lechner et al. [12] Krsek et al. [18] Sobti et al. [21]
p16INK4a	Oropharynx	IHC, tumor	Surrogate HPV biomarker	Routinely used in clinical practice	Gallus et al. [16]
ctHPV DNA	Oropharynx	PCR, plasma	Post-treatment surveillance, accuracy for detecting recurrence	Emerging	Hanna et al. [22]

**Table 2:** DNA methylation biomarkers in HNSCC.

Biomarker	HNSCC Subsite	Detection Method/Specimen	Clinical Use	Current Clinical Status	Study
Differentially methylated regions (ATP5F1EP2, BEND4, C1QL3, CDKN2A, HOXB4, ITGA4, NID2, ZNF137P, ZNF439, ZNF773, ZNF93)	HPV-positive OPSCC	Tumor tissue	Diagnosis	Investigational	Liouta et al. [24].
Zinc finger family biomarkers (ZNF14, ZNF160, ZNF420, ZNF582/PAX1)	HNSCC, particularly OSCC	Tissue, saliva, exfoliated oral epithelial cells	Diagnosis	Investigational	Mao et al. [25]. Mao et al. [25]
Tumor suppressor and carcinogenesis-related genes (DCC, EDNRB, MED15)	HNSCC	Tumor tissue	Cancer risk assessment	Investigational	Liouta et al. [24] Rapado-González et al. [26]
Diagnosis and recurrence biomarkers (HOXA9, PROM1, OPRL1, OPRM1)	HNSCC	Tumor tissue	Early diagnosis and recurrence prediction	Investigational	Liouta et al. [24]
Biomarkers associated with prognosis (hypomethylation of PLAU, MTHFD1L, XPR1)	HNSCC	Tumor tissue	Tumor progression and metastatic potential	Investigational	Liouta et al. [24]
Biomarkers associated with prognosis (hypermethylation of PDCD1, GALR2, SSTR1, SST, COL1A2, DAPK1, TAC1, GALR1, NPY1R, PITX1, C5orf66-AS1, RYR2, PROM1)	HNSCC	Tumor tissue	Poor prognosis	Investigational	Liouta et al. [24]
Biomarkers associated with prognosis (hypomethylation of NID2, RIPOR3, CTLA4, TNFSF11)	HNSCC	Tumor tissue	Favorable prognosis	Investigational	Liouta et al. [24]
Anti-EGFR response biomarkers (EGFR, DAPK)	HNSCC	Tumor tissue	Prediction of anti-EGFR response	Investigational	Liouta et al. [24]
Radiotherapy response biomarkers (CCND1, CCND2, CD47)	HNSCC	Tumor tissue	Prediction of radiotherapy response	Investigational	Liouta et al. [24]
Chemotherapy response biomarkers (CRIP1, G0S2, MLH1, OPN3, S100 family, TUBB2A)	HNSCC	Tumor tissue	Prediction of chemotherapy response	Investigational	Liouta et al. [24]
Chemotherapy response biomarkers (hypermethylation of MGMT)	LSCC	Tumor tissue	Prediction of chemotherapy response	Investigational	Maniaci et al. [27]
Immunotherapy response biomarkers (CD274/PD-L1, GITR, OX40)	HNSCC	Tumor tissue	Prediction of immunotherapy response	Investigational	Liouta et al. [24]

**Table 4:** Proteomic biomarkers in HNSCC.

Biomarker	Category	HNSCC Subsite	Detection Method/Specimen	Clinical Use	Current Clinical Status	Study
EGFR	Growth factor receptor	HNSCC	Tumor tissue	Prognostic biomarker, prediction of response to anti-EGFR therapy, therapeutic target	Clinically validated	Cívico-Ortega et al. [36]
FGFR1	Growth factor receptor	HNSCC	Tumor tissue	Prognostic biomarker; therapeutic target	Emerging	Turri-Zanoni et al. [39]
p53 protein	Tumor suppressor protein	Particularly HPV-negative HNSCC	Tumor tissue	Prognostic biomarker, prediction of chemoradiotherapy response	Emerging	Kang et al. [37]
VEGFA	Vascular growth factor	HNSCC	Tumor tissue	Diagnostic and prognostic biomarker; therapeutic target	Emerging	Alhegaili et al. [38]
TGF	Growth factor	HNSCC	Tumor tissue	Regulation of tumor progression and tumor microenvironment	Emerging	Saini et al. [2]
c-MET	Membrane tyrosine kinase receptor	HNSCC	Tumor tissue	Tumor progression, metastasis, treatment resistance; therapeutic target	Emerging	Raj et al. [41]
Heat shock proteins (HSP27, HSP47, HSP70)	Stress-response proteins	LSCC, OSCC	Tumor tissue	Platinum-based chemotherapy resistance, radioresistance, prognosis	Emerging	Verro et al. [42]
Metallothioneins	Stress-response proteins	HNSCC	Tumor tissue	Tumor progression	Emerging	Verro et al. [42]
Heme oxygenase/Nrf2	Stress-response proteins	HNSCC	Tumor tissue	Chemotherapy resistance, radioresistance	Emerging	Verro et al. [42]
COX-2	Stress-response proteins	HNSCC	Tumor tissue	Tumor progression, recurrence following surgical treatment, metastasis, radioresistance, therapeutic target	Emerging	Verro et al. [42]
Integrin αVβ6, Integrin β8, FAK, Podoplanin	Cell adhesion and invasion proteins	Mainly in OSCC	Tumor tissue	Poor prognosis, lymph node metastasis, tumor invasion	Emerging	Saini et al. [2] Sakurai et al. [43]
E-cadherin, Cyclin D1	Cell adhesion and proliferation proteins	OSCC	Tumor tissue	Early diagnosis	Emerging	Paul et al., Dar et al. [44,45]
Claudin-1, K17, Stathmin	Cell adhesion and proliferation proteins	HPV-positive HNSCC, LSCC	Tumor tissue	Poor prognosis, tumor progression, metastatic potential, therapeutic target (Claudin-1)	Emerging	Lozar et al. [47], Murthy et al. [48], Lopez-Galindo et al. [49], Cavalieri et al. [50]
ALDH	Cancer stem cell biomarker	HNSCC, plasma	Tumor tissue	Poor prognosis, treatment resistance, therapeutic target	Emerging	Maniaci et al. [27], Zanoni et al., Xia et al. [51,52]
DPP-4, Fibrinogen, Cathepsin B, Cornulin, PARK7/DJ-1	Diagnostic protein biomarkers	Mainly OSCC	Saliva, plasma	Diagnosis, early detection, screening	Emerging	Abdel Fattah Tarrad et al. [46], Lopez-Galindo et al. [49], Pillai et al. [54]
MMP-2, MMP-9	Extracellular matrix remodeling proteins	HNSCC	Tumor tissue	Tumor invasion and metastatic dissemination	Emerging	Zou et al. [56]
NTx, CTx	Bone resorption biomarkers	OSCC	Plasma	Prediction of bone invasion and disease progression	Emerging	Boscolo-Rizzo [57]
HIF-1α, HIF-2α, GLUT-1, CA-IX	Hypoxia-associated proteins	HNSCC	Tumor tissue	Prognostic biomarkers and potential therapeutic targets	Emerging	Singh et al., Swartz et al. [58,59]
Perforin-1	Immune-related protein	HNSCC	Tumor tissue	Tumor proliferation and metastasis	Emerging	M et al. [60]
Survivin	Apoptosis-blocking	HNSCC	Tumor tissue	Resistance to oncologic treatments	Emerging	Lopez-Galindo et al. [49]
Ki-67	Cell proliferation biomarker	HNSCC	Tumor tissue	Tumor progression and lymph node metastasis	Emerging	Lopez-Galindo et al. [49]

**Table 5:** Cytokine and chemokine biomarkers in HNSCC.

Biomarker	Category	HNSCC Subsite	Specimen/Detection Method	Clinical Use	Current Clinical Status	Study
IL-6	Cytokine	HNSCC	Serum	Prediction of recurrence, prognosis, prediction of Cetuximab response	Emerging	Saif et al. [61]
CXCL1	Chemokine	OSCC	Tumor tissue	Poor prognosis	Emerging	Saif et al. [61]
CCL2	Chemokine	OSCC	Tumor tissue	Tumor progression, metastatic potential	Emerging	Saif et al. [61]
CCL3/CCL4	Chemokines	OSCC	Tumor tissue	No significant association with carcinogenesis	Not established	Saif et al. [61]
CXCR4	Chemokine receptor	HNSCC	Tumor tissue	Tumor progression, lymph node metastasis, prognosis	Emerging	Saif et al. [61]
CXCR2	Chemokine receptor	HNSCC	Tumor tissue	Prediction of nodal involvement	Emerging	Saif et al. [61]
CXCR3/CXCL9 axis	Chemokine receptor/ligand	OSCC	Tumor tissue	Cervical lymph node metastasis, poor prognosis	Emerging	Saif et al. [61]
IL-10	Cytokine	HPV-positive OSCC	Tumor tissue, saliva	Poor prognosis	Emerging	Saif et al. [61]
IL-17	Cytokine	OSCC	Tumor tissue	Disease progression	Emerging	Jayachandran et al. [62]
XCR1	Chemokine receptor	OSCC	Tumor tissue	Possible role in metastasis	Investigational	Braun et al., Li et al. [63,64]

**Table 6:** Tumor microenvironment biomarkers in HNSCC.

Biomarker	Category	HNSCC Subsite	Specimen/Detection Method	Clinical Role	Current Clinical Status	Study
PD-L1 (CD274)	Immune checkpoint	HNSCC	Tumor tissue; peripheral blood (soluble PD-L1, extracellular vesicles, circulating tumor cells)	Predictive biomarker for response to PD-1 inhibitors; prognosis	Routine clinical use (CPS)	Ascione et al. [65], Sobhaniet al. [67], de Ruiter et al. [68]
PD-1 (CD279)	Immune checkpoint	HNSCC	Tumor-infiltrating immune cells	Target for immunotherapy	Routine clinical use (target)	Ascione et al. [65], de Ruiter et al. [68]
CTLA-4 (CD152)	Immune checkpoint	HNSCC	Activated T cells; regulatory T cells	Target for immunotherapy	Routine clinical use (target)	Park et al. [66], de Ruiter et al. [68]
CD3+CD137+ lymphocytes	Immune cell biomarker	Recurrent/metastatic HNSCC	Peripheral blood	Prediction of response to immune checkpoint inhibitors	Emerging	Ascione et al. [65]
Neutrophil-to-lymphocyte ratio (NLR)	Systemic inflammatory biomarker	HNSCC	Plasma	Prognostic biomarker	Emerging	Sobhaniet al. [67]
Tertiary lymphoid structures (TLS)	Tumor immune microenvironment	HNSCC	Tumor tissue	Favorable prognosis; prediction of immunotherapy response	Emerging	Sobhaniet al. [67]
CD56+ natural killer cells	Immune cell biomarker	HNSCC	Tumor tissue	Prediction of immunotherapy response	Emerging	Sobhaniet al. [67],
Myeloid-derived suppressor cells	Immunosuppressive cells	HNSCC	Tumor tissue	Reduced response to immune checkpoint inhibitors	Emerging	Ascione et al. [65]
M2 tumor-associated macrophages	Immunosuppressive cells	HNSCC	Tumor tissue	Reduced response to immune checkpoint inhibitors	Emerging	Ascione et al. [65]
N2 tumor-associated neutrophils	Immunosuppressive cells	HNSCC	Tumor tissue	Reduced response to immune checkpoint inhibitors	Emerging	Ascione et al. [65]
CD73	Immunoregulatory molecule	HNSCC	Tumor tissue	Reduced responsiveness to immunotherapy	Emerging	Ascione et al. [65]
Type I and II interferons	Cytokines	HNSCC	Tumor microenvironment	Induction of PD-L1 expression	Emerging	Ascione et al. [65]
LAG-3	Immune checkpoint	HNSCC	Tumor-infiltrating lymphocytes	Novel immunotherapeutic target	Emerging	Sobhaniet al. [67]
TIM-3	Immune checkpoint	HNSCC	Tumor-infiltrating lymphocytes	Advanced locoregional disease; immunotherapeutic target; lymph node metastasis	Emerging	Sobhaniet al. [67]
Tumor mutational burden (TMB)	Genomic immune biomarker	HNSCC	Tumor tissue (NGS)	Prediction of immunotherapy response	Emerging	de Oliveira Filho et al. [69]
Microsatellite instability (MSI)	Genomic immune biomarker	HNSCC	Tumor tissue	Prediction of immunotherapy response; platinum resistance	Not routinely recommended in HNSCC	de Oliveira Filho et al. [69] Ascione et al. [65] Sobhaniet al. [67]

**Table 7:** Metabolomics in HNSCC.

Biomarker	Category	HNSCC Subsite	Specimen/Detection Method	Clinical Role	Current Clinical Status	Study
Indoleamine 2,3-dioxygenase (IDO)	Tryptophan metabolism	HNSCC	Tumor tissue	Predictive biomarker of response to immunotherapy; associated with immunotherapy resistance and poor prognosis	Emerging	Saini et al. [2], Ascione et al. [65]
Kynurenine	Tryptophan metabolism	HNSCC	Plasma	Tumor angiogenesis	Emerging	Lin et al. [70]
Sialic acid	Glycosylation biomarker	OSCC	Tumor tissue	Biomarker of malignant transformation; tumor progression and metastatic potential	Emerging	Jayasankar et al. [71]
Malondialdehyde	Oxidative stress biomarker	HNSCC	Tumor tissue, plasma	Diagnosis and disease management	Emerging	Mohideen et al. [72]
Superoxide dismutase (SOD)	Oxidative stress biomarker	OSCC	Tumor tissue	Associated with carcinogenesis	Emerging	Drotarova et al. [73]
Glutathione, glutathione reductase, glutathione peroxidase	Oxidative stress biomarkers	OSCC	Tumor tissue, plasma	Adaptive response to oxidative stress	Emerging	Drotarova et al. [73]
Extracellular ATP	Biomarker of cellular energy metabolism	HNSCC	Tumor tissue	Tumor progression; chemotherapy resistance	Emerging	Zhang et al. [74]
Lactate dehydrogenase (LDH)	Biomarker of cellular energy metabolism	HNSCC	Plasma	Prognostic biomarker; tumor progression, invasion, metastasis, chemotherapy resistance	Emerging	Zhou et al., Barbi et al. [8,75]
Triosephosphate isomerase 1 (TPI1)	Biomarker of cellular energy metabolism	LSCC	Not specified	Potential tumor biomarker	Investigational	Li J Di et al. [76]

**Table 8:** Microbiota in HNSCC.

Biomarker	Category	HNSCC Subsite	Specimen/Detection Method	Clinical Role	Current Clinical Status	Study
Reduced α-diversity, altered β-diversity; reduced Corynebacterium and increased Dialister, Selenomonas, and Streptococcus	Oral microbiome profile	Oral cavity cancer/HNSCC	Oral microbiome analysis	Oral dysbiosis associated with HNSCC and cancer progression	Emerging	Pandey et al. [81]
Fusobacterium nucleatum	Oral bacterium	Oral cavity cancer (more frequent in HPV-positive tumors)	Oral microbiome analysis	p53 suppression, oxidative stress, DNA damage; associated with HPV-positive tumors	Emerging	Urzica et al. [84]
*Porphyromonas gingivalis*	Oral bacterium	Oral cavity cancer	Oral microbiome analysis	p53 suppression, oxidative stress, DNA damage, local tumor invasion, chemotherapy resistance	Emerging	Urzica et al. [84]
*Peptostreptococcus stomatis*, *Haemophilus influenzae*, *Fusobacterium periodonticum*, *Parvimonas micra*, *Streptococcus constellatus*, *Filifactor alocis*, *Pseudomonas aeruginosa*, and *Veillonella* spp.	Oral bacteria	OSCC	Oral microbiome analysis	Increased abundance in OSCC-associated dysbiosis	Emerging	Urzica et al. [84]
Treponema denticola	Oral bacterium	OSCC	Oral microbiome analysis	Tumor invasiveness through dentilisin expression	Emerging	Urzica et al. [84]
Aggregatibacter actinomycetemcomitans	Oral bacterium	Periodontitis-associated OSCC	Oral microbiome analysis	Promotes inflammation, uncontrolled cellular proliferation, and angiogenesis	Emerging	Urzica et al. [84]
*Streptococcus anginosus*	Oral bacterium	HNSCC	Oral microbiome analysis	Acetaldehyde production, genomic instability, activation of IL-8, COX-2 and TNF-α	Emerging	Urzica et al. [84]
*Candida albicans*	Oral fungus	HNSCC	Oral microbiome analysis	Acetaldehyde production; enhanced inflammation, DNA damage, reduced apoptosis, and tumor proliferation when coexisting with *F. nucleatum* or *P. gingivalis*	Emerging	Urzica et al. [84]
Recent antibiotic exposure (particularly fluoroquinolones)	Microbiome-related clinical factor	HNSCC receiving ICIs	Clinical history	Reduced response to immunotherapy and decreased survival	Emerging	Saini et al. [2], Ascione et al. [65]

## Data Availability

All data are available from the corresponding author upon request.

## Supplementary Materials

The following supporting information can be downloaded at: https://www.mdpi.com/article/10.3390/biomedicines14081842/s1, Table S1: PRISMA-ScR-Checklist, Table S2: Studies_included.

## Abbreviations

The following abbreviations are used in this manuscript:

HNSCC	Head and neck squamous cell carcinoma
HNC	Head and neck cancer
HPV	Human papillomavirus
NCCN	National Comprehensive Cancer Network
PCR	Polymerase Chain Reaction
ISH	In situ Hybridization
IHC	Immunohistochemistry
TNM	Tumor, Node, Metastasis
CDKN2A	Cyclin-dependent Kinase Inhibitor 2A
OPSCC	Oropharyngeal Squamous Cell Carcinoma
AJCC	American Joint Committee on Cancer
ICI	Immune checkpoint inhibitor
DCC	Deleted in Colorectal Cancer
ATP5F1EP2	ATP synthase F1 subunit epsilon pseudogene 2
BEND4	BEN domain-containing 4
C1QL3	Complement C1q-like protein 3
CCDC181	Coiled-coil domain-containing 181
CTNND2	Catenin delta-2
CCND1	Cyclin D1
ELMO1	Engulfment and cell motility protein 1
DPP-4	Dipeptidyl peptidase-4
HOXB4	Homeobox B4
HOXA9	Homeobox A9
PROM1	Prominin 1 (CD133)
OPRL1	Opioid receptor-like 1
OPRM1	Opioid receptor mu 1
PLAU	Plasminogen activator, urokinase
MTHFD1L	Methylenetetrahydrofolate dehydrogenase
XPR1	Xenotropic and polytropic retrovirus receptor 1
ITGA4	Integrin subunit alpha 4
NID2	Nidogen 2
OR6S1	Olfactory receptor family 6 subfamily S member 1
SFMBT2	Scm-like with four MBT domains 2
VSTM2B	V-set and transmembrane domain-containing 2B
ZNF	Zinc Finger Protein
PAX	Paired box 1
MED15	Mediator complex subunit 15
GALR	Galanin receptor
SST	Somatostatin
COL1A1	Collagen type I alpha 1 chain
DAPK1	Death-associated protein kinase 1
TAC1	Tachykinin precursor 1
NPY1R	Neuropeptide Y receptor Y1
PITX1	Paired-like homeodomain 1
C5orf66-AS1	Chromosome 5 open reading frame 66 antisense RNA 1
Ryr2	Ryanodine receptor 2
RIPOR3	RHO family-interacting cell polarization regulator 3
TNFSF11	Tumor necrosis factor superfamily member 11
ERBB1	Erb-B2 receptor tyrosine kinase 1
CRIP1	Cysteine-rich protein 1
MLH1	MutL homolog 1
OPN3	Opsin 3
TUBB2A	Tubulin beta 2A class IIa
MGMT	O6-methylguanine-DNA methyltransferase
EDNRB	Endothelin Receptor Type B
EGFR	Epidermal Growth Factor Receptor
TP53	Tumor Suppressor Protein
VEGFA	Vascular Endothelial Growth Factor A
LSCC	Laryngeal Squamous Cell Carcinoma
SOD	Superoxide dismutase
Nrf2	Nuclear Factor Erythroid 2-Related Factor 2
COX-2	Cyclooxygenase-2
ALDH	Aldehyde dehydrogenase
TME	Tumor microenvironment
MMR	Mismatch repair
ATP	Adenosine Triphosphate
MSI	Microsatellite Instability
TIL	Tumor-infiltrating Lymphocytes
PD-1	Programmed cell death protein 1
PD-L1	Programmed Death-Ligand 1
CTLA-4	Cytotoxic T-Lymphocyte Antigen 4
FDA	Food and Drug Administration
EMA	European Medicines Agency
CPS	Combined Positive Score
LAG-3	Lymphocyte Activation Gene 3
TIM-3	T-cell immunoglobulin and mucin-domain-containing 3
CXCL	Chemokine (motif C-X-C) ligand
CCL-2	C-C motif chemokine ligand 2
MMP-9	Matrix metalloproteinase 9
HIF	Hypoxia-Inducible Factor
CA-IX	Carbonic anhydrase IX
GLUT-1	Glucose transporter 1
IDO1	Indoleamine 2,3-dioxygenase 1
FGFR-1	Fibroblast growth factor receptor 1
c-MET	Mesenchymal–epithelial transition factor
HGF	Hepatocyte growth factor
LDH	Lactate dehydrogenase
NADH	Nicotinamide adenine dinucleotide
TPI1	Triosephosphate isomerase 1
