# Supplementary material for: Tumor Biomarkers in Head and Neck Squamous Cell Carcinoma: From Etiology and Pathogenesis to Treatment Response—A Scoping Review
Source: Biomedicines. 2026 Aug 16;14(8):1842. doi: 10.3390/biomedicines14081842 (PMC13509719; doi:10.3390/biomedicines14081842)
Supplement: Supplementary file 1 [file biomedicines-14-01842-s001.zip › Supplementary_Table_S2_Studies_included.pdf]

Supplementary Table S2. Characteristics of the included sources of evidence

Study	Study type/design	Population	Tumor subsite	Biomarker(s)	Outcome / main finding
Saini et al. [2]	Review	Not applicable	HNSCC	Tumor biomarkers	Reviewed biomarkers relevant to precision immunotherapy; multiple biomarkers have diagnostic, prognostic or predictive potential.
Satapathy et al. [10]	Systematic review and meta-analysis	Patients with HNC in India	HNSCC	HPV	Summarized HPV prevalence and clinical relevance in HNC.
Golusiński et al. [11]	Narrative review	Not applicable	HNSCC	HPV	Reviewed HPV-driven HNC and its clinical and molecular characteristics.
Lechner et al. [12]	Narrative review	Not applicable	Oropharynx	HPV	Reviewed epidemiology, molecular biology and clinical management of HPV-associated OPSCC.
Hoffmann et al. [14]	Narrative review	Not applicable	HNSCC	HPV/p16	Reviewed clinical relevance of HPV and p16 in HNC.
Muñoz-Bello et al. [15]	Narrative review	Not applicable	HPV-positive HNSCC	Transcript-based biomarkers	Reviewed potential transcript biomarkers predicting clinical outcomes.
Gallus et al. [16]	Primary study – retrospective/observational	Patients with LSCC	Larynx	p16	Evaluated p16 expression as a surrogate or independent prognostic marker.
Silver et al. [17]	Narrative review	Not applicable	HPV-associated OPSCC	HPV/p16 and treatment-response biomarkers	Reviewed de-escalation strategies and biomarkers relevant to treatment selection.
Krsek et al. [18]	Narrative review	Not applicable	HPV-positive HNC	HPV-related molecular biomarkers	Reviewed advances in diagnostics for HPV-positive HNC.
Mehanna et al. [19]	Narrative review	Not applicable	HPV-mediated HNC	HPV testing biomarkers	Reviewed advances in HPV testing for HNC.
Lorenzo-Pouso et al. [20]	Systematic review and meta-analysis	Patients with oral potentially malignant disorders	Oral cavity	CDKN2A/p16INK4a	Evaluated predictive value of p16 expression for malignant transformation.
Sobti et al. [21]	Narrative review	Not applicable	HNSCC	Radiation-resistance biomarkers	Reviewed predictors of radiation resistance and potential radiosensitizers.
Hanna et al. [22]	Primary study – retrospective/observational	Patients with HPV-driven OPSCC under surveillance	Oropharynx	Circulating TTMV-HPV DNA	Evaluated negative predictive value for surveillance of HPV-driven OPSCC.
Liouta et al. [24]	Narrative/systematic review	Not applicable	HNC	DNA methylation biomarkers	Reviewed diagnostic, prognostic and predictive DNA methylation biomarkers.
Mao et al. [25]	Primary study	Patients with OSCC and controls	Oral cavity	PAX1 and ZNF582 hypermethylation	Evaluated hypermethylation in oral exfoliated cells as a potential OSCC biomarker.
Rapado-González et al. [26]	Systematic review	Patients with oral cancer / controls	Oral cavity	DNA methylation markers	Reviewed methylation markers for oral cancer detection in non- and minimally invasive samples.
Maniaci et al. [27]	Narrative review	Not applicable	Larynx	Gene-expression biomarkers	Reviewed integration of gene-expression biomarkers for

Study	Study type/design	Population	Tumor subsite	Biomarker(s)	Outcome / main finding
					personalized laryngeal cancer treatment.
Komitova et al. [29]	Narrative/critical review	Not applicable	Larynx	miRNAs	Reviewed miRNAs as prognostic biomarkers in laryngeal carcinoma.
Osan et al. [30]	Narrative review	Not applicable	Oral cavity	miRNAs	Reviewed links between miRNAs and oral cancer pathogenesis and their biomarker potential.
Dioguardi et al. [31]	Systematic review and meta-analysis	Patients with oral cancer	Oral cavity	miR-21	High miR-21 expression was associated with poorer overall survival.
Sanesi et al. [32]	Systematic review	Patients with HNSCC	HNSCC	Salivary exosomal miRNAs	Reviewed diagnostic and prognostic potential of salivary exosomal miRNA profiles.
Patel et al. [33]	Primary study	Patients with OSCC	Oral cavity	Salivary exosomal miR-1307-5p	Associated with disease aggressiveness, poor prognosis and chemoresistance.
Jayaraj et al. [34]	Systematic review and meta-analysis	Patients with HNC	HNSCC	miRNAs	Reviewed miRNA expression associated with chemotherapeutic resistance.
Prasad et al. [35]	Narrative review	Not applicable	Oral cavity	Salivary miRNA signatures	Reviewed salivary miRNAs for diagnosis and prognosis of oral cancer.
Cívico-Ortega et al. [36]	Systematic review and meta-analysis	50 primary studies of OSCC	Oral cavity	EGFR	EGFR overexpression associated with poorer survival and adverse clinicopathological features.
Kang et al. [37]	Primary study – retrospective biomarker analysis	179 patients with recurrent/metastatic HNSCC	HNSCC	TP53 mutations	TP53 mutations associated with poorer progression-free and overall survival.
Alhegaili et al. [38]	Primary study – multi-omics/experimental	HNSCC cell lines and public datasets	HNSCC	VEGFA and EGFR	Overexpression associated with advanced disease and reduced survival; potential diagnostic/prognostic relevance.
Turri-Zanoni et al. [39]	Narrative review	Not applicable	Sinonasal cancers	TP53, EGFR, FGFR1, PD-L1, VEGF and other molecular biomarkers	Reviewed molecular biomarkers for diagnosis, prognosis and treatment of sinonasal cancers.
Manzano-Moreno et al. [40]	Narrative review	Not applicable	Oral cavity	Salivary miRNAs and cytokines	Reviewed salivary biomarkers for diagnosis and prognosis of OSCC.
Raj et al. [41]	Narrative review	Not applicable	HNSCC	c-MET/HGF and related pathways	Reviewed c-MET/HGF signaling in proliferation, invasion, angiogenesis and treatment resistance.
Verro et al. [42]	Narrative review	Not applicable	Larynx	Protein/oxidative stress/inflammation biomarkers and miRNAs	Reviewed biomarkers associated with progression, metastasis, recurrence and treatment resistance in LSCC.
Sakurai et al. [43]	Primary study – retrospective observational	96 patients with OSCC	Oral cavity	Integrin αV/β family and FAK/pFAK	pFAK and integrin β8 showed potential prognostic significance.

Study	Study type/design	Population	Tumor subsite	Biomarker(s)	Outcome / main finding
Paul et al. [44]	Narrative review	Not applicable	Oral cavity	Salivary miRNAs, proteins, metabolites, CTCs and ctDNA	Reviewed salivary biomarkers for non-invasive OSCC diagnosis and monitoring.
Dar et al. [45]	Primary study – observational	60 OSCC tissue samples	Oral cavity	E-cadherin and Cyclin D1	E-cadherin decreased and Cyclin D1 increased with increasing tumor grade.
Abdel Fattah Tarrad et al. [46]	Primary study – diagnostic accuracy	45 participants: OSCC, OPMD and controls	Oral cavity	Serum and salivary DPP-4	Evaluated DPP-4 as a potential diagnostic marker for oral cancer and precancerous lesions.
Lozar et al. [47]	Scoping review	Not applicable	HNSCC and other cancers	Keratin 17	Reviewed prognostic and predictive significance of K17.
Murthy et al. [48]	Scoping review	43 original studies	Oral cavity / leukoplakia	Keratin family, especially K17 and K13	Mapped keratin biomarkers in oral leukoplakia and dysplasia.
López-Galindo et al. [49]	Systematic review	10 studies / 1,024 OSCC patients	Oral cavity	Ki-67, HSP60, Survivin, E-cadherin, PD-L1, DPP-4 and others	Reviewed diagnostic, prognostic and therapeutic biomarker evidence in OSCC.
Cavalieri et al. [50]	Primary study – retrospective cohort	100 patients with advanced HNSCC	Oral cavity, oropharynx, hypopharynx, larynx	Claudin-1	Higher CLDN1 expression associated with shorter overall and disease-free survival.
Zanoni et al. [51]	Narrative review	Not applicable	Solid tumors including HNSCC	ALDH isoforms	Reviewed ALDH in cancer stemness, progression and resistance to anticancer therapy.
Xia et al. [52]	Narrative review	Not applicable	Solid tumors	ALDH isoforms	Reviewed ALDH as potential biomarkers and therapeutic targets.
Pillai et al. [53]	Systematic review	Studies of proteomic biomarkers in OSCC	Oral cavity	Proteomic biomarkers	Reviewed proteins identified in tissue, saliva and serum/plasma for diagnosis and prognosis.
Shankavaram et al. [54]	Narrative review	Not applicable	Squamous cell carcinomas including oral/oropharyngeal SCC	Cornulin	Reviewed Cornulin as a potential diagnostic and prognostic biomarker.
Zou et al. [55]	Narrative review	Not applicable	HNC/HNSCC	Protein biomarkers including p53, PTEN, DJ-1, Cornulin	Reviewed current and emerging protein biomarkers for diagnosis and prognosis.
Boscolo-Rizzo et al. [56]	Primary study – retrospective cohort	925 HPV-negative HNSCC patients	HNSCC	NLR, PLR, LMR, SIM, SII	Inflammatory blood indices were associated with overall survival and treatment outcomes.
Singh et al. [57]	Primary study – observational	50 OSCC patients	Oral cavity	Serum NTx and CTx	Elevated NTx/CTx associated with probable early bony microinvasion.
Swartz et al. [58]	Primary study – biomarker analysis	44 patients with laryngeal cancer	Larynx	HIF-1α and pimonidazole	Evaluated tumor hypoxia; neither marker predicted benefit from ARCON over AR.
Indu et al. [59]	Primary study – immunohistochemical	50 OSCC and 50 normal mucosa samples	Oral cavity	HIF-1α, HIF-2α and α-SMA	Expression was higher in OSCC and correlated with cancer-associated fibroblast marker α-SMA.

Study	Study type/design	Population	Tumor subsite	Biomarker(s)	Outcome / main finding
Guan et al. [60]	Narrative review	Not applicable	Cancer including HNSCC	Perforin-1	Reviewed PRF1 in tumor immunity, progression and therapeutic targeting.
Saif et al. [61]	Primary study—retrospective biomarker study	Patients with HNC treated with ICIs	HNSCC	Cytokine-based serum proteomic score	Evaluated a serum cytokine-based score for prognostic stratification and immunotherapy benefit.
Jayachandran et al. [62]	Primary study—comparative observational	Oral cancer, OPMD and healthy controls	Oral cavity	Salivary IL-10 and IL-17	Evaluated cytokines as potential salivary diagnostic biomarkers.
Braun et al. [63]	Systematic review	Studies of OSCC and precursor lesions	Oral cavity	Chemokines and chemokine receptors	Reviewed diagnostic, prognostic and metastatic significance of chemokines.
Li et al. [64]	Narrative review	Not applicable	Oral cavity / OSCC	Chemokines and chemokine receptors	Reviewed roles in proliferation, invasion, angiogenesis, immune regulation and metastasis.
Ascione et al. [65]	Narrative review	Not applicable	Recurrent/metastatic HNSCC	PD-L1, PD-1, CTLA-4, TMB, MSI, TILs and immune biomarkers	Reviewed biomarkers for immunotherapy selection; PD-L1/CPS remains the principal clinically used predictive biomarker.
Park & Skålhegg [66]	Narrative review	Not applicable	Multiple cancers including HNSCC	PD-1, PD-L1 and CTLA-4	Reviewed combination checkpoint inhibition and the need for predictive biomarkers.
Sobhani et al. [67]	Narrative review	Not applicable	HNC/HNSCC	PD-L1, PD-1, CTLA-4, LAG-3, TIM-3, TMB, TILs and others	Reviewed immune biomarkers for prognosis and immunotherapy response.
de Ruiter et al. [68]	Primary study – retrospective comparison	147 HNSCC patients	HNSCC	PD-L1, assessed by 22C3, SP263 and laboratory-developed assays; TPS/CPS	Demonstrated moderate-to-poor concordance among PD-L1 assays, emphasizing assay standardization.
de Oliveira Filho et al. [69]	Primary study—retrospective observational	Patients with OPSCC	Oropharynx	MLH1, MSH2, MSH6 and PMS2	Evaluated MMR protein expression and its potential biomarker relevance.
Lin et al. [70]	Primary study—experimental/translational	28 HNSCC tissue samples plus experimental models	HNSCC	Kynurenine, IDO1, mTOR and angiogenesis markers	Kynurenine promoted angiogenesis through IDO1/mTOR signaling.
Jayasankar et al. [71]	Systematic review and meta-analysis	22 studies; 14 meta-analyzed	Oral cavity	Salivary sialic acid	Salivary sialic acid was higher in oral cancer/precancer; potential early detection biomarker, but no standardized cutoff.
Mohideen et al. [72]	Systematic review and meta-analysis	46 studies; 26 meta-analyzed	Oral cavity / OSCC	Malondialdehyde	MDA was generally increased in plasma, serum and saliva in OSCC.
Drotárová et al. [73]	Primary study—case report	2 patients with oral white lesions	Oral cavity	SOD, miR-17, miR-23a, miR-206 and antioxidant markers	Observed altered SOD/miRNA profiles associated with malignant transformation; evidence limited by two cases.
Zhang et al. [74]	Narrative review	Not applicable	HNSCC	Immunogenic cell death biomarkers	Reviewed ICD-related biomarkers in chemoresistance and immunoregulation.
Barbi & Purohit [75]	Systematic review and meta-analysis	9 studies	Oral cavity / OPMD	Serum LDH	Serum LDH was higher in potentially malignant disorders than healthy controls.

Study	Study type/design	Population	Tumor subsite	Biomarker(s)	Outcome / main finding
Li et al. [76]	Primary study—multicenter observational/translational	82 LSCC and 56 non-tumor tissue specimens plus external datasets	Larynx	TPI1	TPI1 was increased in LSCC and showed diagnostic/prognostic potential.
Srivastava et al. [79]	Review	Not applicable	HNSCC	Oral microbiome / dysbiosis-associated taxa	Reviewed the role of oral dysbiosis in HNSCC development and progression.
Pandey et al. [81]	Primary study—observational microbiome study	Patients with HNC and controls	HNSCC / head and neck cancer	Oral microbiome composition and bacterial taxa	Demonstrated site-dependent microbial differences and emphasized standardized sampling.
Urzică et al. [84]	Narrative review	Not applicable	Oral cavity / oral cancer	Oral microbiome and microbiota-associated pathways	Reviewed interactions between oral microbiome and oral cancer pathogenesis and biomarker potential.
